# MicroRNAs in *Daphnia magna* identified and characterized by deep sequencing, genome mapping and manual curation

**DOI:** 10.1038/s41598-019-52387-z

**Published:** 2019-11-04

**Authors:** Dag H. Coucheron, Marcin W. Wojewodzic, Thomas Bøhn

**Affiliations:** 1 MIRG, Department of Medical Biology, UiT The Arctic University of Norway, N-9037 Tromsø, Norway; 2 School of Biosciences, University of Birmingham, Edgbaston, Birmingham, B15 2TT UK; 3 Research Department, Cancer Registry of Norway, Ullernchausseen 64, 0379 Oslo, Norway; 4 Institute of Marine Research, PB 6404, N-9294 Tromsø, Norway

**Keywords:** High-throughput screening, miRNAs

## Abstract

MicroRNAs (miRNAs) are small non-coding RNAs that function in RNA silencing and post-transcriptional regulation of gene expression in most organisms. The water flea, *Daphnia magna* is a key model to study phenotypic, physiological and genomic responses to environmental cues and miRNAs can potentially mediate these responses. By using deep sequencing, genome mapping and manual curations, we have characterised the miRNAome of *D. magna*. We identified 66 conserved miRNAs and 13 novel miRNAs; all of these were found in the three studied life stages of *D. magna* (juveniles, subadults, adults), but with variation in expression levels between stages. Forty-one of the miRNAs were clustered into 13 genome clusters also present in the *D. pulex* genome. Most miRNAs contained sequence variants (isomiRs). The highest expressed isomiRs were 3′ template variants with one nucleotide deletion or 3′ non-template variants with addition of A or U at the 3′ end. We also identified offset RNAs (moRs) and loop RNAs (loRs). Our work extends the base for further work on all species (miRNA, isomiRs, moRNAs, loRNAs) of the miRNAome of *Daphnia* as biomarkers in response to chemical substances and environment cues, and underline age dependency.

## Introduction

Non-coding, regulatory small RNAs constitute one of the epigenetic mechanisms of gene regulation. These regulatory RNA molecules are evolutionary conserved across phyla, and elements are found in a vast majority of organisms from bacteria to animals and even viruses^1–4^. The main types of small RNAs in animals are microRNA (miRNA), piwi-interacting RNAs (piRNAs) and small interfering RNAs (siRNAs)^5^. Of these, miRNAs have been the most studied group and are found to regulate a massive number of biological processes by interacting with mRNAs and non-coding RNAs. The canonical miRNA genes are transcribed mainly by RNA polymerase II into a primary transcript called pri-miRNA. Pri-miRNAs are processed by the DROSHA/Pasha (Pasha is known as DGCR8 in mammals) microprocessor complex to precursor miRNAs (pre-miRNAs) (~70 nucleotides) which are then transported to cytoplasm by Exportin 5/Ran-GTP and further processed by Dicer to ~22 bp duplex sequence with 2 nucleotides overhang at the 3′ end. Usually either the 5′- or the 3′-end (5p or 3p arm) of the pre-miRNA is chosen as mature guide miRNA when associated with Argonaute (Ago) proteins in a functional RNA-induced silencing complex (RISC) that is the regulatory unit. The other strand (passenger strand or miRNA*) may be discarded^5,6^. However, arm switching may occur where the dominant guide miRNA shifts from one arm to the other of the pre-miRNA across different tissues, developmental stages and between animal species^7,8^. The pri-miRNA and pre-miRNA transcripts may undergo alternative cleavage by Drosha and Dicer and thereby produce sequence variants with different start and end positions compared to the mature miRNAs (3′ - and 5′ template variants). Sequence variants may also be produced by nucleotide additions (3′- and 5′-end) and single nucleotide substitutions (SNPs) (non-template variants). All these variants of mature miRNA are called isomiRs and seem to be functional and have regulatory properties^8–10^. The nucleotides of position 2–7 from the 5′ end of the mature guide miRNA form the seed sequence that recognize and bind to the target sequences. The binding may also involve nucleotide eight and to a lesser extent nucleotide at position 13–16^11^. Adding or deleting nucleotides at the 5′ end and SNPs in the seed sequence of the mature miRNA will change the seed sequence and may give different targeting properties^12^. Previous deep sequencing projects have also revealed reads from miRNA offset RNAs (called moRs) of the pri-miRNA transcript adjacent to the 5′ end of mature miRNA of the 5p arm and to the 3′ end of mature miRNAs of the 3p arm^13,14^. Reads have also been identified from the loop of pre-miRNAs^15,16^ and these loop-derived sequences are named loRs^17^. The functions of moRs and loRs are still unclear.

MiRNAs are prevalent in insects^18^ and are involved in regulation of several biological processes^19^, as for example insect physiology^20^ like metamorphosis^21^ or immune response^22^ and even targeting viruses^23^.

*Daphnia magna* and *D. pulex* are the aquatic key invertebrate model organisms in ecotoxicology, ecology and evolution^24^. Responses to different triggers and stressors, including environmental pollutants, pesticides and GMOs, have been studied on its life-history and physiology as well as on a genetic and epigenetic levels^25–29^. Recently, some attention has been given to describe miRNAs in the genus *Daphnia*. In *D. pulex*, 229 microRNAs were identified *in silico* and verified by qPCR^30^ and microarrays^31^ while in *D. magna* 205 putative mature miRNAs were identified based on small RNA sequencing and mapping against databases^32^. Eighteen of these were confirmed by secondary structure analysis of miRNA hairpins. Recently, Hearn *et al*.^33^ reported 33 miRNAs in *D. magna* by deep sequencing and genome mapping. The authors also investigated whether transgenerational effect could be mediated via miRNAs and showed that miRNAs played a role in maternal provisioning rather than longer-term transgenerational responses. Indeed, the expression profiles of miRNA are a promising step to further examine organism responses to stressors, in particular on specific genes, genetic pathways or networks^34^. Thus, miRNAs may extend their use as important biomarkers^31,33,35^. However, it has recently been reported that annotating miRNAs without mapping sequenced reads to the same species genome may largely overestimate the presence of miRNAs^36^. For instance, Yalle *et al*.^36^ found that insects contained 65 conserved families with very low variation.

In this study, we characterize the miRNAome from three different life stages (juveniles, subadults, adults) in *D. magna* by a four step process: 1) deep sequencing of small RNAs, 2) alignment of the trimmed, clean reads to the *D. magna* genome, 3) identification of putative miRNA by aligning the mapped reads to miRBase and 4) manual inspection of sequence and reads in the genome at the position of the putative miRNA. With this approach we aimed at improving the coverage and verification of authentic presence of this important class of gene regulating nucleic acids, building on previous miRNA studies in *D. magna*^32,33^. We identify conserved and novel miRNAs, miRNA clusters, isomiRs, offset RNAs (moRs) and loop RNAs (loRs), and compare these data between juveniles, subadults and adults. Our work presents novel miRNA discoveries and characterisation on age-specific miRNAs in *Daphnia* which can be used for further verification and investigation.

## Results and Discussion

### Analyses of the small RNA sequencing data

The raw reads from deep sequencing of three small RNA libraries, one each from the three life stages of pooled *D. magna* juveniles, subadults and adults, were processed into trimmed clean reads of 17–30 nucleotides (Table 1).

**Table 1:** Overview of the handling of reads (not normalized) from Illumina sequencing of small RNAs from three developmental stages of *D. magna* (juveniles, subadults, adults). 1) nt: nucleotides; 2) Percentage of “Clean reads 17–30 nt after trimming”; 3) Percentage of “Clean reads 17–30 nt mapped to *D. magna* genome”; 4) Percentage of mapped reads annotated.

	Juvenile	Subadult	Adult
Raw reads	20,031,228	18,722,856	19,245,107
Clean reads	19,499,693	18,300,832	18,574,516
Clean reads 17–30 nt^1)^ after trimming	18,099,382 (92.8%)^2)^	17,093,022 (93.4%)^2)^	17,280,988 (93.0%)^2)^
Clean reads 17–30 nt^1)^ mapped *D. magna* genome	17,391,597 (96.1%)^3)^	16,539,316 (96.8%)^3)^	16,443,140 (95.2%)^3)^
Mapped reads annotated from miRBase and dpu-miRs	6,159,000 (35.4%)^4)^	2,505,931 (15.2%)^4)^	6,037,898 (36.7%)^4)^

The trimmed clean reads were mapped to the genome of *D. magna* and about 96% of the clean reads of 17–30 nucleotides from all three libraries aligned to the genome sequence (Table 1).

**Figure 1:**
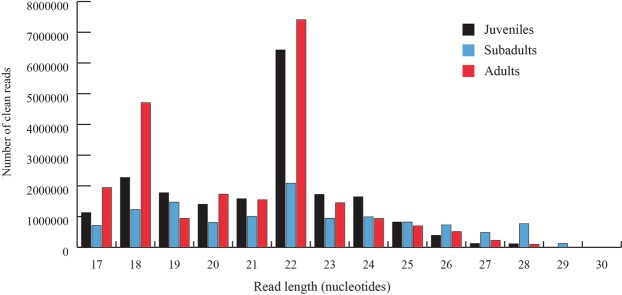
Read counts distributed on read lengths for sequenced small RNA from juvenile, subadult and adult *D. magna*, mapped to its genome (normalized read counts).

As a first step to identify miRNAs, reads mapping to the *D. magna* genome were aligned to miRNAs in miRBase release 21^37^ and dpu-miRNAs from *D. pulex*^30^. The percentage of mapped reads that aligned to miRNA sequences was similar for juveniles (35.4%) and adults (36.7%) but only 15.2% for subadults (Table 1). The mapped read counts were normalized and distributed on read lengths (Fig. 1). For all three life-stages, reads of 22 nucleotides were most abundant, but the number of reads of 22 nucleotides from subadults was less than one third of the reads of 22 nucleotides from juveniles and adults. The next highest counts were found for reads of 18 nucleotides (juveniles, adults) and 19 nucleotides (subadults). Overall, juveniles and adults had more read counts of lengths below 24 nucleotides, while subadults had more reads with lengths between 25–30 nucleotides (Fig. 1).

### Identification of conserved miRNAs in *D. magna*

**Figure 2:**
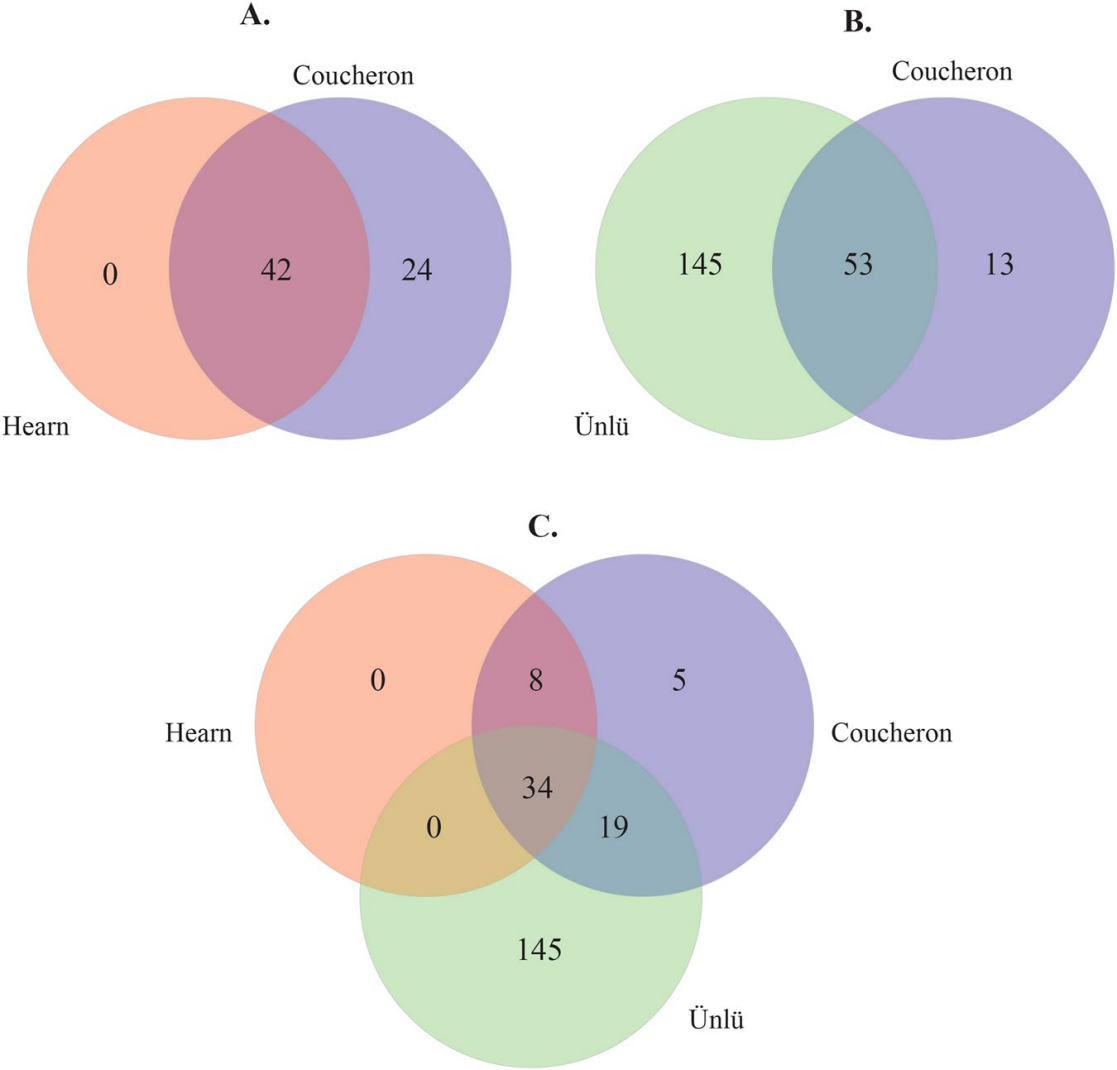
Venn diagram showing the number of overlapping miRNAs from Ünlü *et al*.^32^, Hearn *et al*.^33^ and this report (Coucheron *et al*.).

The reads that mapped to the *D. magna* genome aligned to several hundred putative miRNA species in miR Base and dpu-miRNAs. To verify that the putative miRNAs identified in miRBase and dpu-miRNAs were real *D. magna* miRNAs, we mapped the mature miRNA sequence of each putative miRNAs back to the *D. magna* genome. The positions in the genome, where each of these mature miRNA sequences aligned, were then manually scanned for sequence and mapped reads in the same regions. Through these analyses we identified the presence of reads that could be annotated to 66 conserved microRNAs in miRBase and dpu-miRNAs, representing 51 miRNA-families (Table 2). This number of miRNA families is similar to a recent re-evaluation of the number of miRNAs in insects, which identified 65 conserved miRNA families^36^. Hearn *et al*.^33^ identified 72 precursor miRNAs in *D. magna* of which all the 33 annotated and nine non-annotated miRNAs were present among our 66 miRNAs. Of the 205 mature sequences representing 188 miRNAs and the 18 miRNA hairpin sequences identified by Ünlü *et al*.^32^ we found 53 miRNA and 17 hairpin sequences in our data. (Fig. 2, Supplementary2 Table S1). Overall, about 52% (34 of 66) of the miRNAs reported by Hearn *et al*.^33^, Ünlü *et al*.^32^ and us overlap (Fig. 2, see Supplementary2 Table S1 for details on the overlap of miRNAs in previous studies^32,33^ and this study). In addition 37 miRNAs of the 229 found in *D. pulex*^30,31^ were identified in *D. magna* in this work (Supplementary2 Table S1).

**Table 2:** The 66 conserved miRNAs and the number of reads (normalized) identified in *D. magna* juveniles, subadults and adults.

miRNA	miRNA family	miRNA hairpins			miRNA	miRNA family	miRNA hairpins		
		Juvenile read counts	Subadult read counts	Adult read counts			Juvenile read counts	Subadult read counts	Adult read counts
mir-1	MIR-1	3221163	3155203	2594699	mir-2a-1	MIR-2	5198	2945	2243
let-7	LET-7	424752	667761	855758	mir-263b	MIR-263	4886	3024	2839
mir-184	MIR-184	194617	289556	508306	mir-305	MIR-305	2839	4573	4360
mir-31	MIR-31	148273	133631	47515	mir-3791	MIR-3791	76	451	3822
mir-375	MIR-375	19497	48431	145329	mir-750	MIR-750	2270	2637	3815
mir-276	MIR-276	130370	96493	109537	mir-252b	MIR-252	2695	3293	3419
mir-263a	MIR-263	71608	73390	30325	mir-92a	MIR-25	3207	2511	2942
mir-275	MIR-275	65173	67463	72255	mir-190	MIR-190	1951	1657	3041
mir-8	MIR-8	7596	8248	59111	mir-279e	MIR-279	401	397	2962
mir-2b	MIR-2	48232	28871	23633	mir-252a	MIR-252	2550	2184	713
mir-279d	MIR-279	44763	39266	38042	mir-745	MIR-745	441	2379	622
mir-317	MIR-317	17301	30606	6061	mir-13	MIR-2	2166	1303	396
mir-71	MIR-71	25624	16333	11908	mir-33	MIR-33	1186	720	2164
mir-279c	MIR-279	25051	25040	19945	mir-283	MIR-216	1590	1326	1794
mir-279a	MIR-279	20845	11446	8564	mir-2944	MIR-2944	238	153	1759
mir-34	MIR-34	6965	19642	2198	mir-281	MIR-46	1040	1218	1461
mir-10	MIR-10	10347	18559	12245	mir-92b	MIR-25	1098	900	1389
mir-124	MIR-124	11038	5404	3424	mir-277	MIR-277	703	1098	804
mir-87–2	MIR-87	4862	10238	3676	mir-965	MIR-965	760	695	1081
mir-87–1	MIR-87	4766	9981	3589	mir-7	MIR-7	643	1030	486
mir-125	MIR-10	4795	6321	9744	mir-998	MIR-998	52	99	834
bantam	BANTAM	7103	8691	6551	mir-210	MIR-210	712	378	161
mir-315	MIR-315	8250	6008	5551	mir-193	MIR-193	398	641	672
mir-2a-2	MIR-2	7316	4149	6069	mir-282	MIR-282	403	527	142
mir-279b	MIR-279	497	817	6748	mir-309	MIR-3	4	48	320
mir-1175	MIR-1175	2495	5994	4257	mir-137	MIR-137	262	139	36
mir-9b	MIR-9	648	709	5970	mir-278	MIR-278	231	182	126
mir-9a	MIR-9	5284	4662	5938	mir-iab-4	MIR-iab-4	225	93	97
mir-100	MIR-10	3037	5280	5761	mir-307	MIR-67	26	17	116
mir-285	MIR-29	4194	4529	5538	mir-iab-8	MIR-iab-8	102	37	37
mir-12	MIR-12	5449	4844	4309	mir-981	MIR-981	75	54	24
mir-993	MIR-10	2939	2130	5208	mir-219	MIR-219	6	8	15
mir-133	MIR-133	2945	5200	1167	mir-153	MIR-153	9	8	5

The number of normalized reads aligned to the miRNA hairpins (pre-miRNA + adjacent sequences) ranged from a few reads (miR-153) to more than 3 million (miR-1) (Table 2). When comparing the miRNA expressions in the three life stages, the adults had highest expression of 28 miRNA hairpins, followed by juveniles with highest expression of 24. Subadults had highest expression of only 14 miRNA hairpins (Table 2).

**Figure 3:**
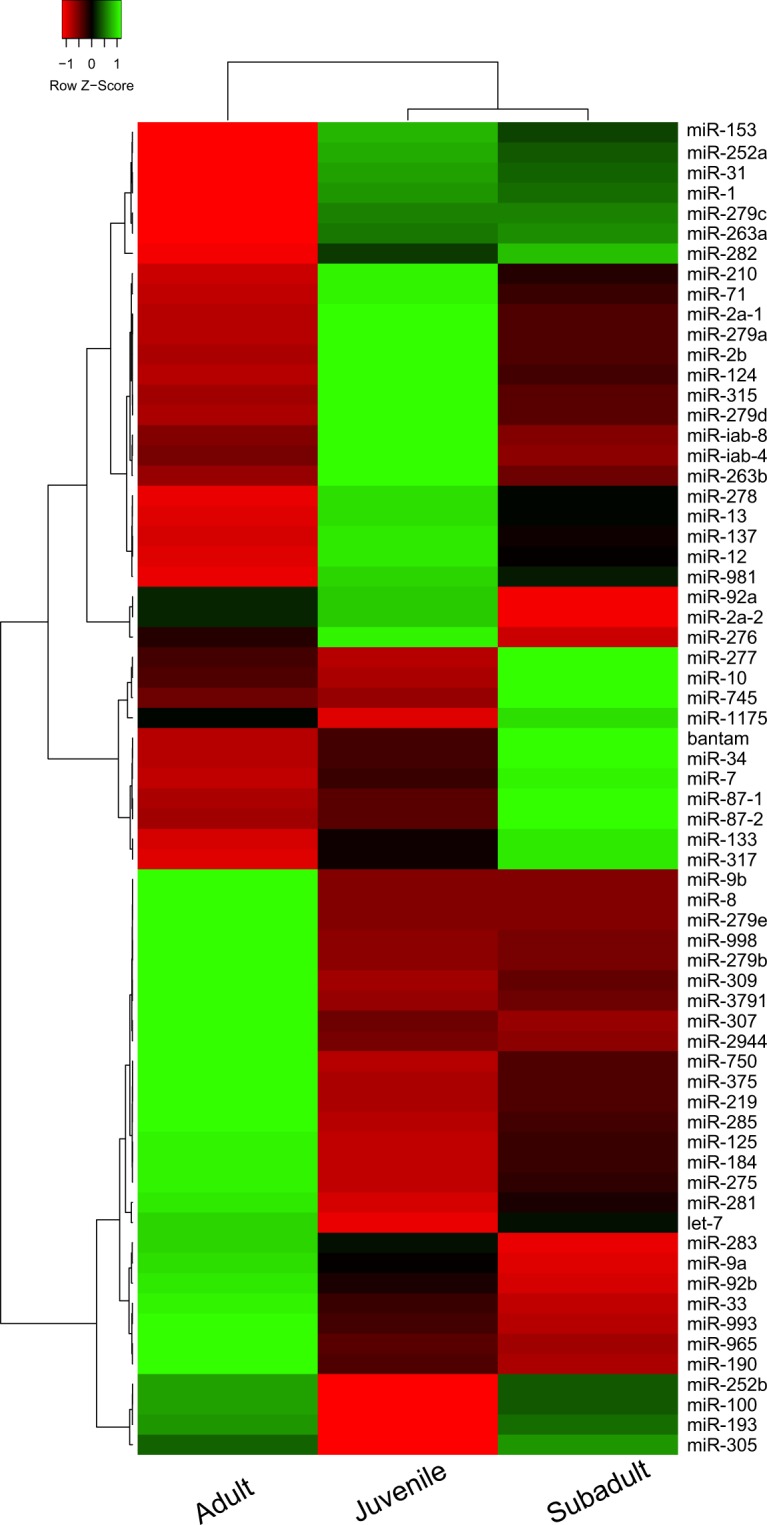
Heat map and clustering of conserved microRNA hairpins from juvenile, subadult and adult *D. magna*, based on normalized read counts. Clustering of the three life stages is shown at the top of the heat map, while clustering of the miRNAs is shown to the left. The clustering was performed by using Pearson distance measurement and average linkage methods.

Notably, the sequenced reads from the 66 miRNA hairpins were from whole animals, thus lacking further details on how different body parts, organs, etc., differ in expression. However, the three age groups expressed all the same 66 species of miRNA, but with some proportional differences between the three different life stages (Fig. 3, Table 2). The heat map (Fig. 3) also depicts some larger groups of miRNAs where the expression pattern of miRNAs in each group was similar across the three life stages. Cluster analysis of the total miRNA read counts of each life stages indicated that juveniles and subadults were the two most similar whereas the adults deviated from these two (Fig. 3).

**Figure 4:**
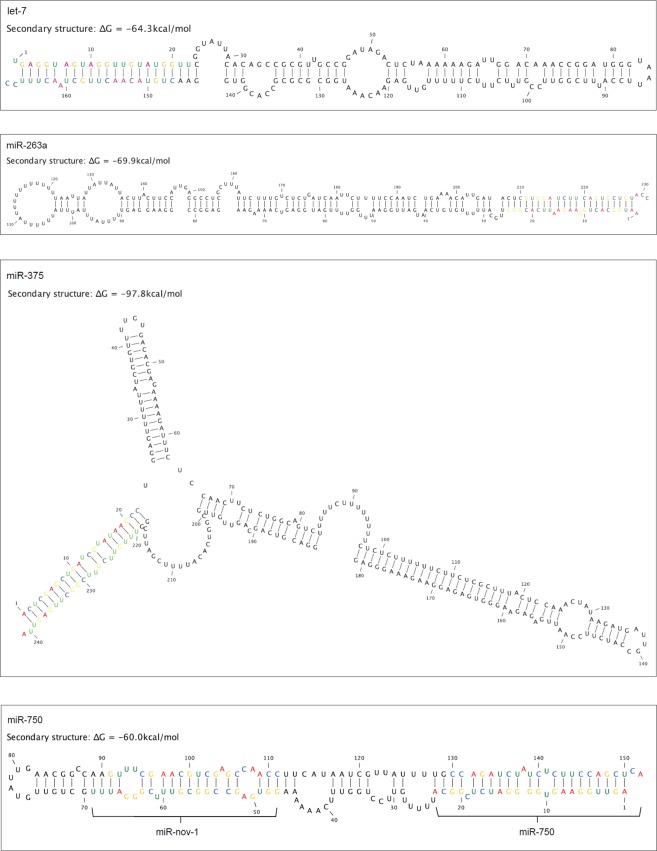
Secondary structure of *D. magna* pre-let-7, pre-mir-263a, pre-mir-375 and pre-mir-750 (coloured nucleotides are the mature miRNAs). The folding free energy (ΔG) is given for each predicted secondary structure of the pre-miRNA.

Most of the conserved pre-miRNAs were between 57–100 nucleotides in length. However, four miRNAs differed with longer lengths: pre-let-7 (168 nucleotides), pre-miR-263a (231 nucleotides), pre-miR375 (241 nucleotides) and pre-miR-750 (152 nucleotides) (Fig. 4). Using the mature miRNA sequences from *D. magna* to map the *D. pulex* genome, we found similar length for dpu-pre-let-7 (179 nucleotides), dpu-pre-miR-375 (282 nucleotides) and dpu-pre-mir-750 (153 nucleotides), while dpu-pre-mir-263a was shorter (92 nucleotides). An interesting observation is that the *D. magna* pre-mir-750 contains a putative novel miRNA inside the mature miR-750-5p and -3p (see below). In the pre-miR-750 of *D. pulex* a putative novel pre-miR hairpin with a rather strong folding free energy^37^ of ∆G < −20 kcal/mol could be detected, but with a different sequence than in *D. magna*.

### Conserved mature miRNAs

Mature guiding strands of miRNAs can be expressed from the 5p arm, 3p arm or both in the pre-miRNAs by changing from one to the other arm in different tissue in the same organism^7,38^. Usually the pre-miRNA arm containing the mature miRNA sequence with the highest read counts is treated as the mature guide miRNA, leaving the sequence of the other strand as passenger (or star) miRNA^5,39^. Sometimes miRNA sequences form both pre-miRNA arms are expressed as mature guide miRNAs and may co-mature due to different expression in different cells (so called arm switching)^38^. In this study, with the primary aim to discover new, and strengthen the description and verification of previously described miRNA in *D. magna*, we did a single sequencing of small RNAs from each of three life stages (relatively large, pooled batches of whole-body juveniles, subadults and adults).

Based on the counts of read sequences and folding of pre-miRNAs, we could identify the mature miRNA sequences from both arms of the vast majority of the conserved miRNA hairpins (Supplementary1 Table S2, Supplementary1 Fig. S1). It should be noted that for the identical mature miRNAs, the reads were equally divided between miR-2a-1-3p and miR-2a-2-3p, and between miR-87-1-3p and miR-87-2-3p due to the impossibility of knowing which miRNA the reads mapped to.

The majority of the mature guide miRNA sequences in *D. magna* were expressed from the 3p arm (44 miRNA) of the pre-miRNA (Supplementary1 Table S2). Almost all of the mature miRNA sequences had more than 50% of the total read counts mapped to its arm of the miRNA hairpin (Supplementary1 Table S3), but with variation from 0% (miR-279b-5p) to 94.6% (miR-2944-5p) (Supplementary2 Table S1). In addition to the read counts of each mature miRNA, additional read counts to each arm were mainly due to miRNA isoforms (isomiRs) (Supplementary2 Table S1. See also below). Although for most of the miRNAs the mature guide miRNA had much more reads than the passenger miRNA, several of the passengers also exhibited rather high read counts (Supplementary1 Table S2). This could indicate that some of these passenger miRNAs may be active regulatory species. However, for a few miRNAs the read counts of mature miRNA sequences from both 5p and 3p arms were similar. To indicate co-expression of mature miRNA from both arms, we used the ratio of <2 between the read counts of the most expressed arm divided by the number of reads from the less expressed arm^36^. We found that some of the mature miRNAs displayed co-expression of both arms in different combinations of juveniles, subadults and adults as shown in Table 3. Furthermore, some mature miRNAs had undergone arm-switching of the most expressed miRNA from one arm to the other in the different life stages of *D magna* (Table 3, Supplementary1 Table S4). The co-expression and arm-switching for miR-2a-1 and miR-2a-2 are highly uncertain since the expression levels of mature miRNAs were only estimated to be equal (see above). We also found that some miRNAs displayed shift in expression between the mature miRNA and its isomiR in the three life stages (see below).

**Table 3:** Mature miRNA co-expressed from the 5p- and 3p-arm (+) and showing arm-switching (highest expressed arm is indicated as 3p or 5p).

Mature miRNA with co-expressed 5p- and 3p arms and arm-switching	Life stages of *D. magna* with co-expressed arms (+) and arm-switching		
	Juvenile	Subadult	Adult
miR-2a-2	+	+	
	3p	3p	5p
miR-750	+	+	
	5p	3p	3p
miR-8	+	+	
miR-125	+		+
miR-282	+		+
	5p	5p	3p
miR-283	+		
miR-965	+		
	3p	3p	5p
miR-2944		+	
	3p	5p	3p
miR-2a-1			+
	3p	3p	5p

The mature guide miRNA sequences of *D*. *magna* were identical or similar to most of the conserved mature guide miRNAs in miR Base, release 21^37^. The mature guide miRNAs (highest expression) are shown in Supplementary1 Table S2, and their putative differential expression between the three developmental stages is displayed in the heat map in Supplementary1 Fig. S2. The clustering of the mature miRNA profiles (Supplementary1 Fig. S2) indicate a closer relationship between juveniles and subadults, as was seen for the miRNA hairpin profiles (Fig. 3). Again, we observed some groups of miRNAs where the pattern of expression in each group was similar across juveniles, subadults and adults. It is interesting to observe that miR-1, let-7, miR-184, miR-275 and miR-276 which are among the highest expressed miRNAs in *D. magna*, are also among the highest expressed miRNAs in the closely related, white shrimp *Litopenaeus vannamei*^40^. In contrast, only miR-1 and miR-276 (and then miR-276-5p and not miR-276-3p which is the highest in our result) were among the five highest expressed *D. magna* miRNAs analysed by Hearn *et al*.^33^. Many of the conserved miRNAs found in *D. magna* have been revealed to have diverse and important biological functions in insects^18,19,21,36^. For instance, let-7 is the most known miRNA that regulates developmental timing in animals. Mir-1, predominantly expressed in muscle tissue, in insects, is responsible for maintaining muscles integrity and mir-184 is involved in regulation of oogenesis^19^.

### Identification of novel miRNAs

**Figure 5:**
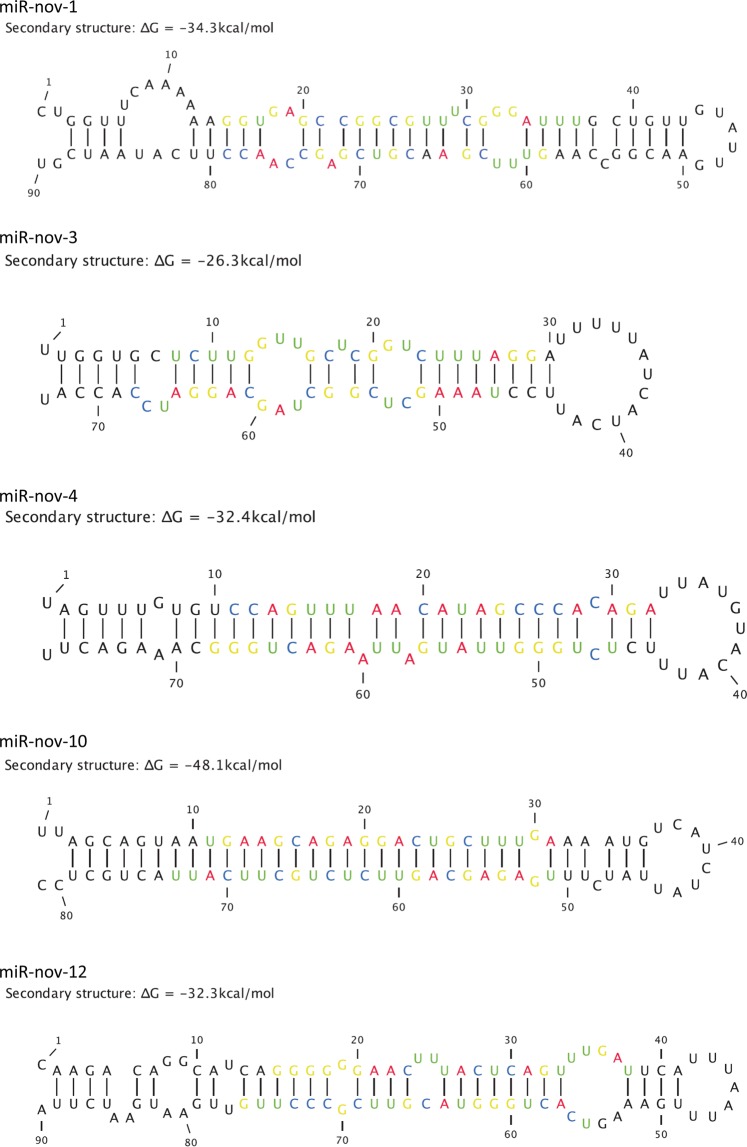
Secondary structure of hairpins of putative novel miRNAs. The folding free energy (∆G) for each hairpin is shown.

Many miRNAs are clustered in the genome due to the formation of new miRNAs close to pre-existing ones^41,42^. In mammalian species, most of the miRNA genes (about 2/3) are clustered within 50 kb^43^. Based on this knowledge we first performed a query for putative novel miRNAs in *D. magna* by manual scanning of scaffolds and contigs that contained the conserved miRNAs. Using the criteria for annotation of novel miRNA^37^ described in the Methods section we identified 13 putative novel miRNAs (Supplementary1 Table S5) of which six were identical with non-annotated precursor miRNAs in *D.magna* reported by Hearn *et al*.^33^ (Supplementary2 Table S1). Five (miR-nov-1, miR-nov-3, miR-nov-4, miR-nov-10 and miR-nov-12) fulfilled the criteria of novel miRNAs in *D. magna* (Table 4). The secondary structures of these five miRNA hairpins with their folding free energies (∆G) are shown in Fig. 5.

**Table 4:** Novel miRNAs with sequence, number of normalized reads and nucleotide (nt) length in juvenile, subadult and adult *D. magna*.

miRNA	Mature miRNA sequence	Reads of mature miRNA			Length (nt)
		Juvenile	Subadult	Adult	
miR-nov-1–5p	GGUGAGCCGGCGUUUCGGGAUUU	9	6	1	23
miR-nov-1-3p	GUUUCGAACGUCGAGCCAACC	43	40	3	21
miR-nov-3-5p	UCUUGGUUGCUCGGUCUUUAGG	21	79	829	22
miR-nov-3-3p	UAAAGCUCGGCUAGCAGGAUCC	20	356	3017	22
miR-nov-4-5p	CCAGUUUAACAUAGCCCACAGA	1	0	9	22
miR-nov-4-3p	UCUGGGUUAUGAUUAAGACUGGG	181	847	4718	23
miR-nov-10-5p	UGAAGCAGAGGACUGCUUUGA	19	73	62	21
miR-nov-10-3p	UGAGAGCAGUUCUCUGCUUCAUU	0	2	3	23
miR-nov-12-5p	GGGGGGAACUUUACUCAGUUUGAU	1	2	0	24
miR-nov-12-3p	UCACUGGGUACGUUCGCCCUUG	2	17	60	22

In addition, eight other pre-miRNAs fulfilled several of the criteria for the annotation as novel miRNAs^37^. However, for most of them mature miRNA reads were only detected from the putative miRNA sequence on one of the hairpin arms (Supplementary1 Table S5, Supplementary1 Fig. S3).

Comparisons of the mature sequences of miR-nov-7-3p, miR-nov-8-3p and miR-nov-9-3p shows that the seed sequence (nucleotide 2-7) is identical in all three (Supplementary1 Table S5). Thus, these miRNAs are of the same family. Several of the novel miRNAs showed a lower expression level in the juveniles compared to subadults and adults, i.e. miR-nov-3-3p, mR-nov-4-3p, miR-nov-12-3p, miR-nov-2-3p, miR-nov-7-3p, miR-nov-9-3p and miR-nov-11-5p) (Table 4, Supplementary1 Table S5). We also observed a tendency of increased expression of some novel miRNAs from juvenile to subadult and to adult stages (e.g. miR-nov-3-3p, miR-nov-4-3p, miR-nov-2-3p). These differently expressed novel miRNAs in different age-classes indicate important roles in the ontogenetic development of *D. magna*, but further studies need to investigate this specifically.

Mapping the *D. pulex* genome with the mature sequence of the novel *D. magna* miRNAs showed that several of these miRNA sequences were also present in *D. pulex* (Supplementary1 Table S6).

Ikeda *et al*.^44^ reported that 21 of the novel miRNAs in *Triops cacriformis* (tadpole shrimp) showed more than 80% sequence similarities with the genomic sequence of these miRNAs in *D. pulex*. In our data we found only four of the *T. cancriformis* novel miRNAs as expressed miRNAs in *D. magna*. It is interesting that the three novel *D. magna* miRNAs: miR-nov-7-3p, miR-nov-8-3p and miR-nov-9-3p have sequences identical to tca-miR-n504-3p, tca-mir-n512-3p and tca-miR-n503-3p from *T. cancriformis*. Moreover, miR-nov-13 and tca-miR-3477 have identical seed sequences, but with five mismatches outside the seed region.

### Genomic clustering of miRNAs

MiRNAs are often found as clusters in metazoan genomes. In flies and worms about 30% of all miRNAs are clustered as two or more miRNAs within 10 kb, while ~45% of conserved miRNAs are found in clusters in these species (and mammals)^42^. Moreover, many of these miRNAs clustered within 10 kb, and especially when clustered within 1 kb, are co-expressed^45–48^. In *D. melanogaster*, miRNAs separated by less than 1 kb seem to be highly co-expressed in different tissues^49^.

Identification of clustered miRNAs (both conserved and novel) within 10 kb of *D. magna* genome and comparison with clustering of the same miRNAs in the *D. pulex* genome, is shown in Supplementary1 Table S7. Both species contain the same 13 clusters with the same order of the miRNAs in each cluster. Of the 66 conserved miRNAs in *D. magna*, 31 (47%) are grouped into 11 clusters. Furthermore, miR-nov-7, miR-8, and miR-nov-9, which are clustered in *D. magna*, have their identical miRNAs tca-miR-n504, tca-miR-n512 and tca-miR-504 clustered in the same orientation in *T. cancriformis*^44^. We also note that miR-283, miR-nov-13, miR-12 build a cluster in *D. pulex*, but these miRNAs are on different scaffolds in *D. magna*. However, scaffold sc1551 (miRNA-283) may be connected to scaffold sc2703 (miR-nov-13, miR-12) since the Thyroid receptor-interacting protein (http://arthropods.eugenes.org:8091/gbrowse/cgi-bin/gbrowse/daphnia_magna7/) is encoded across these two scaffolds. Thus, these three miRNAs seem to be within a 10 kb distance also in *D. magna*. Another support for this cluster is that similar clusters are documented in other organisms. For example, the tca-miR-12/miR-3477 (same seed as miR-nov-13)/miR-283 cluster is within 1000 bp in *T. cancriformis*^44^, the miR12/miR-1889/miR-283 in *Anopheles gambiae*^50^, and similar clusters in several other arthropods (see miRBase).

Several miRNA clusters are conserved among animals^42^. In mammals, clustered miRNAs may function as a unit in regulation of biological processes^51^. Many of the *D. magna* (and *D. pulex*) clusters are found as similar or identical clusters in other arthropods (miRBase release 21^37^). Particularly many of the *D. magna* clusters are conserved in *T. cancriformis* (10 clusters)^44^, *Tribolium castaneum* (9 clusters)^52^ and *Apis mellifera* (9 clusters) (miRBase release 21^37^). Among the *D. magna* clusters with the highest expressed miRNAs are the miR-100/let-7/miR*-*125 cluster which have been found conserved in all metazoans^53^ and is involved in regulating the larvae-to-adult transition as well as neural development^54^. The miR-2/miR-13/miR-71 cluster is conserved in many invertebrates^55^ and was recently reported to be involved in resistance to the insecticide deltamethrin in mosquitos^56^.

### miRNA isoforms (isomiRs) in *D. magna*

**Figure 6:**
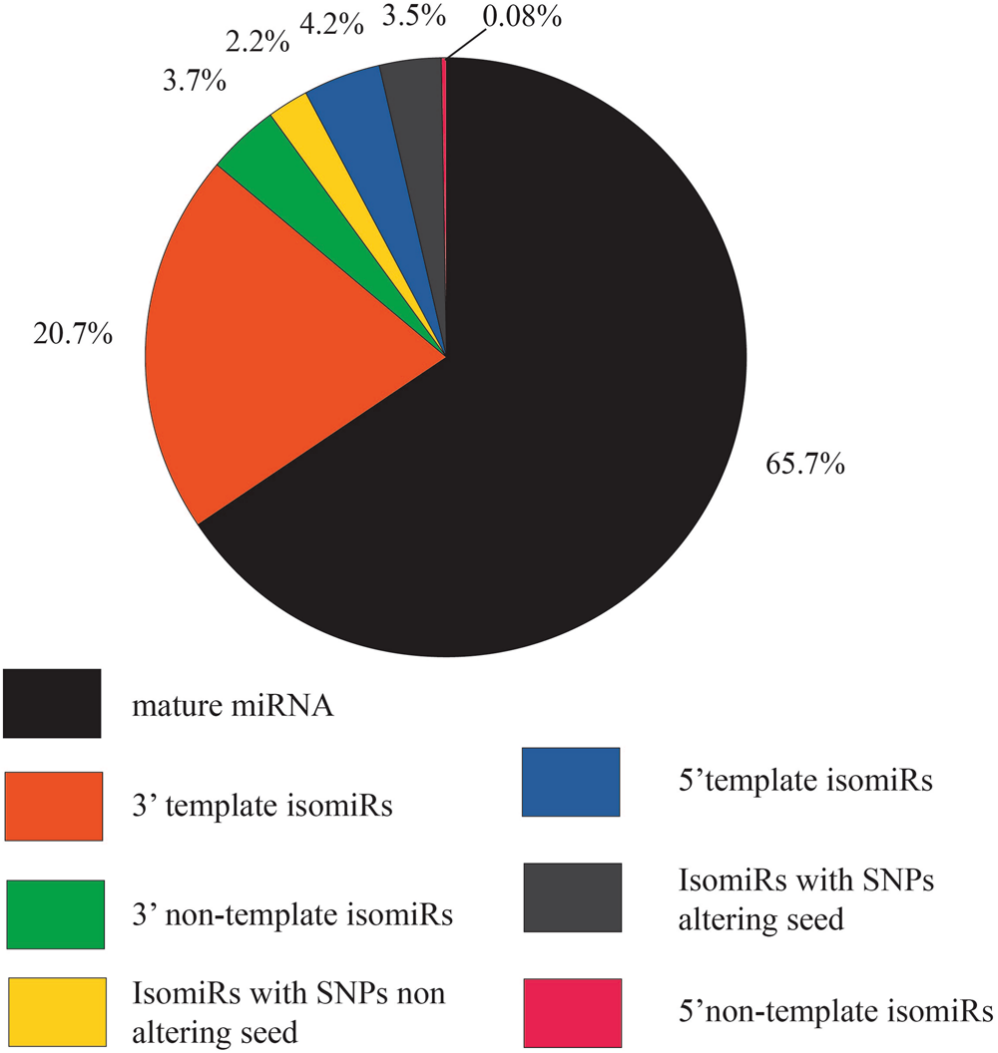
Mean percentage ratio of mature miRNAs and isomiR reads out of total read counts for 5p + 3p arms.

Deep sequencing of small RNAs has shown that most miRNAs are expressed both as mature sequences and as variants (isomiRs)^8,9,57^. We have analysed the read sequences that mapped with more than 10 read counts to miRNA hairpins. The read sequences have been divided into seven groups: i) mature miRNAs; ii) 3′ template isomiRs containing reads with the mature 5′ end and deletions or additions at the 3′ end complementary to the genome sequence; iii) 3′ non-template isomiRs containing reads with mature 5′ end and 3′ additions not complementary to the genome sequence; iv) isomiR reads not part of groups ii) or iii) and with the mature 5′ end but with single nucleotide polymorphisms (SNPs) not altering the mature seed sequence; v) 5′ template isomiRs with reads containing the mature 3′ end and deletions or additions at the 5′ end complementary to the genome sequence; vi) 5′ non-template isomiRs containing reads with intact mature 3′ end and additions at the 5′ end not complementary to the genome sequence; and vii) isomiR reads not part of group v) or vi) and with SNPs altering the seed sequence^10^. Although the majority of *D. magna* read counts were mature miRNA sequences (conserved and novel), expressed in all the three developmental stages (juveniles, subadults, adults), all miRNA hairpin arms (5p or 3p) mapped with more than 10 reads, contained isomiRs (Supplementary2 Table S1). For each miRNA calculation of the ratio of read counts for each of the seven isomiR groups, divided on the total read count for the miRNA arm, showed large variation both for each miRNA and each isomiR group (Supplementary2 Table S1). Moreover, determining the mean ratio for each isomiR group (across all miRNAs) revealed large variation also in the sizes of each isomiR group. Generally, the highest expressed isomiRs were the 3′-templated variants (mean 20.7% of the total read counts for 5p+3p arms). The mean level of expression of the other variants was as following: 3′ non-template isomiRs, 3.7%; 5′ template isomiRs, 4.2%; isomiR reads with SNPs changing seed sequence, 3.5%; isomiR reads with SNPs not altering seed, 2.2% and 5′ non-template isomiRs, 0.08%. (Fig. 6, Supplementary1 Table S8). Supplementary1 Figure S4a & b) shows mean level of expression for each arm. Although some of these variants displaying one or a few reads may be sequencing errors, most isomiR sequences have several read counts and are present in all three independent sequencing samples (juvenile, subadult, adult stages) supporting that they are real isomiR sequences.

Interestingly, the proportional relationship between the different isomiR variants in *D. magna* is similar to that reported for the feline (cat family) miRNAome^58^, suggesting that the roles and functions of mature miRNAs and their isomiRs may be conserved by evolution, even across distantly related organisms. A recent review suggests that isomiRs may function as an additional repression tool beside the mature miRNA^59^. For some miRNAs we found 3′ template variants that had a similar level of expression as their mature miRNAs: miR-1175-5p (two variants), miR-12-5p (mainly one variant), miR-279b-3p (juveniles, mainly one variant), miR-71-5p (mainly three variants), miR-993-5p (mainly three variants), miR-998-3p (juveniles and subadults, one variant; adults, two variants), miR-iab-8-5p (juveniles and adults, two variants; subadults, three variants), miR-9b-5p (two variants), miR-31-5p (one variant), miR-34-5p (mainly three variants), miR-210-5p (one variant), miR-285-3p (mainly one variant), miR-2944-3p (one variant), miR-317-3p (mainly two variants), miR-92b-3p (one variant), miR-981-3p (one variant) and 5′ template variants: miR-210-3p, miR-305-3p, miR-9a-3p (summarised in Table 5). In some cases the level of expression between mature miRNA and one of its 3′ template or 5′ template isomiR variants were noticeably different between the three developmental stages, e.g miR-12-5p, miR-210, miR-285-3p, miR-31-5p miR-998-3p, miR-305-3p, miR-9a-3p (Table 5). MiR-71-5p was special in this regard since the 3′ template variants had from 55% to 120% more read counts than the mature sequence (Supplementary2 Table S1, Table 5). However, the 3′ template isomiRs reads are here mainly divided on three different sequences with one to three deletions at the end (Table 5).

**Table 5:** IsomiR variants with similar read counts as the mature miRNA (^*^)putative mature passenger strand. (Not normalized read counts).

Mature miRNA isomiR	Sequence	Juvenile read counts	Subadult read counts	Adult read counts
miR-1175-5p*^)^	AAGUGGAGCAGUGGAUC	305	143	250
isomiR 3′ template	AAGUGGAGCAGUGGAUCU	249	114	214
miR-12-5p	UGAGUAUUACAUCAGGUACUGGU	3293	1002	2405
isomiR 3′ template	UGAGUAUUACAUCAGGUACUGG	3035	1090	2389
miR-210-5p*^)^	AGCUGCUGAACACUGCCCAAGAU	58	6	14
isomiR 3′ template	AGCUGCUGAACACUGCCCAAGA	62	5	11
miR-279b-3p	UGACUAGAACCCACACUCGUCCGG	266	323	5801
isomiR 3′ template	UGACUAGAACCCACACUCGUCCG	155	22	173
miR-285-3p	UAGCACCAUUGGAAUUCAGUUU	2722	1239	2879
isomiR 3′ template	UAGCACCAUUGGAAUUCAGUUUA	2436	872	3678
miR-2944-3p	UAUCACAGUCGUAGUUACUAGA	131	32	1693
isomiR 3′ template	UAUCACAGUCGUAGUUACUAG	121	6	247
miR-31-5p	AGGCAAGAUGUCGGCAUAGCUGA	77395	22752	27715
isomiR 3′ template	AGGCAAGAUGUCGGCAUAGCUG	77848	27525	20359
miR-71-5p	UGAAAGACAUGGGUAGUGAGAUGU	8911	3063	4268
isomiR 3′ template	UGAAAGACAUGGGUAGUGAGAUG	7710	1371	2434
	UGAAAGACAUGGGUAGUGAGAU	7303	1357	3802
	UGAAAGACAUGGGUAGUGAGA	7022	1723	2934
miR-9b-5p	UCUUUGGUGGUCUAGCUGUAUGA	92	170	3094
isomiR 3′ template	UCUUUGGUGGUCUAGCUGUAUG	228	77	2504
	UCUUUGGUGGUCUAGCUGUAU	308	53	1070
miR-9b-3p*^)^	UAAAGCUAGAUCAGCAAGGCAA	20	18	263
isomiR 3′ template	UAAAGCUAGAUCAGCAAGGCA	34	5	53
miR-92b-3p	AAUUGCACUCGUCCCGGCCUGC	629	131	1022
isomiR 3′ template	AAUUGCACUCGUCCCGGCCUG	602	220	514
miR-998-3p	AUAGCACCACGGGAUUCAGCCGC	11	15	317
isomiR 3′ template	AUAGCACCACGGGAUUCAGCCG	36	19	168
miR-210-3p	CUUGUGCGUGUGACAGCGGCUAU	378	64	85
isomiR 5′ template	UUGUGCGUGUGACAGCGGCUAU	308	85	62
miR-305-3p*^)^	CGGCACCUGCUGGAGUGCAAUUG	311	81	267
isomiR 5′ template	CACCUGCUGGAGUGCAAUUG	125	129	326
miR-9a-3p*^)^	AUAAAGCUAGGUUACCAAAGUUA	164	40	84
isomiR 5′ template	UAAAGCUAGGUUACCAAAGUUA	146	63	97

Although 3′ template isomiR variants usually are the most common, very little is known about their functions. In a recent report Yu *et al*.^60^ found that 3′ template isomiRs of miR-222 could play functional roles in human cell lines. We found that the highest expressed 3′ template variants were usually those with one deletion at the end (Supplementary 2 Table S1). However, some exceptions were also found where the 3′ end additions were non-template A or U (see below).

Non-templated 3′ variants generated by post-transcriptional uridylation or adenylation have been found to change stability, affect expression and target selection^43,61,62^. In our data the highest read counts were 3′ non-template variants with A or U additions at the end and some of these had expression values similar to 3′ template variants, e.g. isomiRs from let-7-5p, miR-1-3p, miR-100-5p, miR-275-3p, miR-8-3p, miR-965-3p. In a few cases the most highly expressed isomiR sequence were a 3′ non-templated one, e.g. let-7-5p, miR-100-5p, miR-13-3p, miR-184-3p, miR-277-3p, miR-34-3p, miR-8-3p (Supplementary2 Table S1).

5′ variants of isomiRs had a mean expression of 4.2% for the 5′ template and only 0.08% for the 5′ non-template variants, respectively (Fig. 6, Supplementary1 Table S8). IsomiRs of the 5′ template type were the highest expressed isomiRs of miR-1175-3p, miR-125-3p, miR-210-3p, miR-252a-3p, miR-275-5p, miR-279c-5p, miR-305-3p, miR-71-3p and miR-9a-3p. However, it is interesting to notice that miR-9a-3p, miR-210-3p, and miR-305-3p displayed 5′ template read counts at a similar level as the potential mature miRNA, and that the most highly expressed sequence of mature miRNA and its 5′ template isomiRs may differ between the three developmental stages (Table 5, Supplementary2 Table S1). Especially the 5′ template variant of miR-1175-3p showed a different pattern of expression (percentage ratio of total read counts) which was low in juveniles (12%) and adults (5%) but high in subadults (41%) (Suplementary2 Table S9a). The same sequence variants of the 5′ template of miR-1175-3p are present in all three life stages although the read counts of each sequence vary between the stages (Suplementary2 Table S9a). Of the isomiRs that displayed seed shifting, the 5′ template isomiRs were generally higher expressed than the 5′ non-template sequences. Since 5′ variants shift the seed sequence they have been found to regulate other RNA sequences than the mature miRNA^12,63^. However, recently it was reported that 5′ variants also may be able to regulate the same mRNA as the mature miRNA^64^.

We observed a mean expression of 3.5% of reads with SNPs that alter the seed sequence and a mean expression of 2.2% of reads with SNPs that did not affect the seed sequence (Fig. 6, Supplementary 1 Table S8). Although some of these SNP variants may be sequencing errors, we find that several variants have the same mismatch in identical sequences with several read counts across the three independent sequencing samples (juvenile, subadult, adult) supporting that these are real isomiRs (Suplementary2 Table S1). The SNP variants with altered seed sequences were the highest expressed isomiRs of miR-190-3p, miR-279c-5p, and miR-750-5p (Suplementary2 Table S9b). Editing of miRNA may result in SNPs both altering the seed sequence and the sequence outside the seed region^65,66^.

Taken together our sequencing results show expression of a variety of isomiRs of miRNAs in *D. magna*. The observed proportions for all three life stages shows that the 3′ variants and variants with SNPs that do not alter the seed, have a mean percentage of 26.5% while the 5′ variants and variants with SNPs changing the seed have a mean percentage of 7.8% (Supplementary1 Table S8). The 3′ variants show the highest expression for most of the miRNAs while the 5′ variants are expressed to a lower degree. IsomiRs have been suggested as promising, additional biomarkers in diagnostic assays based on mature miRNA for studying illness such as for example cancers^67,68^. In *D. magna* isomiRs may be developed into important biomarkers in addition to the role of mature miRNAs for analysing biological changes.

### Offset- and loop miRNAs

Analysis of reads adjacent and in the loop of pre-miRNAs revealed one or more reads which were recognised as moRs and/or loRs (Supplementary2 Table S10). Nine miRNAs display moRs and/or loRS with more than 10 reads in at least one the three life stages of *D. magna* (Table 6). MiR-7 is interesting since it contains 5′ moR reads that add up to relatively high percentages of the mature miR-7 reads (juveniles: 27%, subadults: 14% and adults: 96%) (Supplementary2 Table S10). As reported previously^17^, we also found that moRs at the 5′ site (5p) of mature miRNAs are more frequent and with higher read counts than on the 3′ site (3p) (Supplementary2 Table S10). In the three investigated life stages the total expressions of loRs were at similar level as for 5′ moRs. However, the expression level of the sum of 5′moR + 3′moR was somewhat higher than the expression of loRs (Supplementary2 Table S10). Both moRs and loRs may play functional roles^15,69^.

**Table 6:** moRs and loRs of miRNAs with more than ten reads in at least one life stage (not normalized read counts).

	moR 5p counts			moR 3p counts			loR counts		
miRNA	Juvenile	Subadult	Adult	Juvenile	Subadult	Adult	Juvenile	Subadult	Adult
let-7	6	4	16	0	0	1	29	8	1
mir-1175	10	12	6	0	2	0	0	0	0
mir-2a-2	0	1	0	0	0	0	13	5	24
mir-278	0	0	0	0	0	0	10	0	10
mir-279a	0	0	1	16	4	37	1	1	2
mir-283	4	1	12	0	0	0	45	14	80
mir-34	17	18	39	0	0	0	6	4	13
mir-7	109	40	224	0	0	0	0	0	1
mir-745	0	0	0	0	0	0	24	25	64

## Conclusions

*Daphnia magna* is an important model organism for ecotoxicology, ecology and evolution. Its remarkable ability to respond to changing environmental conditions, i.e. its plasticity, could potentially be mediated via age-specific miRNA expression. As such, miRNAs could constitute an important epigenetic mechanism in clonal organisms. Age or life-stage in an animal represent an important organismal context and needs to be considered for future experimental designs whenever miRNA is of interest. This is also in agreement with previous work^33^.

The main purpose of this study was to characterise the miRNAome and thereby identify and validate the number of miRNAs in *D. magna* by deep sequencing, mapping of reads to its genome combined with manual curation of putative miRNAs. By this procedure we identified and further characterized 66 conserved and 13 novel miRNAs from three life stages of *D. magna* (the pre-miRNA sequences are shown in Supplementary3 Table 11). Of the 66 miRNAs we found, 42 were already reported by Hearn *et al*.^33^ and 17 miRNA hairpins and 53 miRNA were already identified by Ünlü *et al*.^31^ All the miRNAs we found were expressed in all the three life stages and displayed similar miRNA profiles. However, clustering of the three miRNA profiles indicated a closer relationship between juveniles and subadults. The mature guide miRNAs were generally expressed with the highest read counts (>50% of total read counts). The additional reads were sequence variants (isomiRs) which were expressed by almost all miRNAs. We identified isomiRs which were 3′-, 5′- and SNP- variants. Several miRNAs contained 3′ template- or 5′ template variants of isomiRs that had a similar expression levels as their mature miRNAs. Furthermore, we found that miRNAs with the highest expression levels could shift between the mature miRNA and one of its 3′ template -or 5′ template isomiR variants over the three life stages. Several miRNAs displayed reads adjacent (offset RNAs (moRs)) and in the loop (loRs) of the pre-miRNAs.

Our work extends the background for further work on using both mature miRNAs and their sequence variants (isomiRs) as biomarkers of stress in *Daphnia* and should also help to exclude age-related confounding in follow-up studies. For future studies we recommend: i) independent validation of miRNAs in other laboratories, where at least 20 million reads is necessary (since we see some differences in our data to previous reports), ii) linked analyses of mRNA and miRNA expression to reveal more of the functional roles of miRNA in general, iii) as complete and detailed as possible reporting of the atlas of miRNA expression in different organs and sub-systems in the organism, also at different stages of development, and iv) test individual miRNAs for their ability and sensitivity to function as biomarkers of different types.

## Materials and Methods

### Experimental setup

All individuals of *D. magna* used in the experiments were from the same clonal population. Animals were kept in M7 medium^70^, fed *Desmodesmus subspicatus*, *ad libitum* and never crowded. The juveniles (n = 120) that were sequenced were born within 24 h from the third clutch of a set of n = 25 mothers. The subadults (n = 40) were also born within 24 hours from a set of n = 25 mothers and reared for 6 days. We checked for visible eggs in the brood chamber of each individual and confirmed that eggs were not present. For the adults, we selected animals with visible eggs at day 9 (n = 30). During the course of the experiment the subadult and adult groups were fed *Desmodesmus subspicatus* green algae with 0.15 mg Carbon per animal per day.

Every third day, we transferred each animal to a new glass with fresh M7 medium. Temperature was held constant at 22 + /− 1.5 degrees C. Light regime was 16 hours of light and 8 hours of darkness. Throughout the experiment, the average pH of the medium was 7.8, oxygen saturation always >95% and average conductivity 575 µs cm^−1^.

### Total RNA extraction

*D. magna* animals were filtered from the growth cultures. For each life stage (juveniles, subadults and adults), 100 mg of animals were added to 1000 µl of Trizol (Invitrogen) and homogenized by bead milling with ceramic beads (Roche Applied Science, Basel, Switzerland) in a Precellys homogenizer (Bertin instruments). The Trizol extraction of total RNA was according to the manufacturer’s protocol with modifications involving prolonged precipitation and centrifugation steps to ensure recovery of the small RNA fraction. Purified total RNAs were dissolved in nuclease free water and stored at −70 °C. RNA quantity was determined with a NanoDrop spectrophotometer (NanoDrop Technologies). The quality of RNA was analysed with an Agilent 2100 Bioanalyzer using an Agilent RNA 600 Nano kit (Agilent Technologies).

### Construction of small RNA library and Illumina sequencing

Total RNAs were shipped on dry ice to the Norwegian Sequencing Center (NSC), Ullevål University Hospital, Oslo (http://www.sequencing.uio.no/) for small RNA library construction and Illumina sequencing. The small RNA libraries were prepared as described in the “Small RNA Sample Preparation Guide (Illumina, Cat # FC 930-1004, Part # 1004239 Rev B, August 2009). After PCR amplification gel-purified small cDNA libraries (fragment size about 100 bp) were sequenced on an Illumina Genome Analyzer.

### Analyses of sequenced reads

Three datasets containing de-multiplexed, clean reads (reads with adaptor clipped off and removed non-clipped reads, adaptor-only reads, N reads, reads < 10 nucleotides) were received from NSC as fastaq files. These clean reads were analysed on a CLC Genomics Workbench (Qiagen) using the following pipeline: 1) The clean reads were trimmed to 17–30 nucleotides (“Trimmed Clean reads”). 2) The trimmed reads were mapped to the *D. magna* genome (dmagna-v2.4-20100422-assembly (http://wfleabase.org/)^71^ using “Map Reads to Reference” module and following mapping parameters: mismatch costs 2, insertion cost and deletion cost each 3, length fraction 0.85, similarity fraction 0.8, map randomly. Reads mapped to *D. magna* genome were then aligned to miRNAs in miRBase release 21 (http://www.mirbase.org)^37^ and *in-silico* determined dpu-miRNAs^30^ using the various “Mature length variants” and “Alignment settings” parameters. The DNA sequence of potential *D. magna* miRNAs recognised from miRBase and dpu-miRNAs alignments (matches) were mapped to the *D. magna* genome and manually inspection of sequence and reads in the genome at the position of the putative miRNA was used to identify and annotate miRNAs. Pre-miRNA sequences were manually identified and secondary structures and their folding free energy (ΔG) of miRNA hairpins were determined by using the “Predict Secondary Structure RNA” parameter in the CLC software.

For examination of the presence of *D. magna* miRNAs in *D. pulex*, miRNA sequences were mapped to the *D. pulex* genome (http://www.ftp://ftp.ensemblgenomes.org/pub/release-34/metazoa/fasta/daphnia_pulex/dna/)^72^

Annotating novel *D. magna* miRNAs was performed mainly by the criteria described previously^17,37,73^ except that we also included putative novel miRNA with reads aligning only to one of the pre-miRNA arms.

MoRNAs were defined as reads that were immediately adjacent either to the 5′ end of mature miRNA of the 5′ arm and/or adjacent to the 3′ end of the mature miRNA of the 3′ arm^14^. LoRNAs were annotated as reads between mature miRNAs of the 5′ and 3′ arm^15^. For both moR and loR we also included reads that started outside the mature miRNA and ended inside the miRNA and which could not be regarded as isomiRs.

We used DESeq2 package (R package) to normalise the raw read counts as described previously^74^. Shortly, as input the DESeq2 package expects raw count data obtained from sequencing of miRNA. The DESeq2 model internally corrects for differences in library size (*estimateSizeFactor function*) and delivers normalized values. Normalized counts were accessed by “counts” function on recalculated counts object. We did not perform statistics to look for significant differences in miRNA expression between the groups due to our experimental design (one independent library per age class). Hierarchical clustering, presented as heat maps, were generated by Heatmapper^75^ using average linkage and Pearson’s distant methods.

## Supplementary information

Supplementary1 informatin

Supplementary2 information

Supplementary3 information

## Data Availability

The data sets supporting the results of this article are included within the article and its supplementary information files. Fastaq files of clean raw reads are uploaded to ENA under our Bioproject accession number PRJEB29358.
