# Supplementary material for: MicroRNAs in Daphnia magna identified and characterized by deep sequencing, genome mapping and manual curation
Source: Sci Rep. 2019 Nov 4;9:15945. doi: 10.1038/s41598-019-52387-z (PMC6828783; doi:10.1038/s41598-019-52387-z)
Supplement: Supplementary file 1 — Supplementary1 informatin [file 41598_2019_52387_MOESM1_ESM.pdf]

MicroRNAs in *Daphnia magna* identified and characterized by deep sequencing, genome mapping and manual curation.

Dag H. Coucheron[1*)], Marcin W. Wojewodzic[2,3*)] & Thomas Bøhn[4*)]

[1)] MIRG, Department of Medical Biology, UiT The Arctic University of Norway, N-9037 Tromsø, Norway

E-mail: dag.coucheron@uit.no

Tel. +47 77644657

[2)] School of Biosciences, University of Birmingham, Edgbaston, Birmingham, B15 2TT UK

E-mail: M.Wojewodzic@bham.ac.uk

[3)] Research Department, Cancer Registry of Norway, Ullernchausseen 64, 0379, Oslo, Norway

E-mail: Marcin.Wojewodzic@kreftregisteret.no

[4)] Institute of Marine Research, PB 6404, N-9294 Tromsø, Norway

E-mail: thomas.bohn@hi.no

Tel. +47 97009916

*) The correspondence can be addressed to all authors

Supplementary1 Information.

Supplementary1 Table S2

Mature miRNA	Juvenile	Subadult	Adult	Order of mature guide miRNA and passenger miRNA
miR-1-3p	2835291	2591831	1854466	1
let-7-5p	355124	569022	605619	2
miR-184-3p	150801	212185	315095	3
miR-276-3p	118549	82513	81331	4
miR-375-3p	12194	28589	85920	5
miR-31-5p	56713	48770	18173	6
miR-263a-5p	46485	51844	18392	7
miR-275-3p	42857	40149	37230	8
miR-2b-3p	31117	16475	13379	9
miR-279d-3p	29986	25522	20212	10
miR-8-5p	3067	3473	26504	11
miR-279a-3p	17559	8782	6044	12
miR-10-5p	7000	16915	7519	13
miR-279c-3p	15278	16011	8199	14
miR-317-3p	9760	13043	2442	15
miR-8-3p	2388	2349	12105	16
miR-87-1-3p	4311	8828	2633	17
miR-87-2-3p	4312	8815	2640	18
miR-124-3p	6774	3029	2136	19
miR-71-5p	6530	6566	2799	20
bantam-3p	5325	5478	4078	21
miR-315-5p	5376	3567	2991	22
miR-125-5p	2220	4375	3640	23
miR-133-3p	2012	4016	629	24
miR-100-5p	2108	3916	3541	25
miR-279b-3p	202	617	3704	26
miR-2a-2-5p	2283	1415	3700	27
miR-993-3p	2351	1428	3613	28
miR-9a-5p	3222	3157	3263	29
miR-2a-1-3p	3234	1695	523	30
miR-2a-2-3p	3233	1711	524	31
miR-279c-5p	2315	1848	3204	32*)
miR-34-5p	3149	6238	831	33
miR-125-3p	1814	996	3032	34
miR-305-5p	1722	2915	2208	35
miR-92a-3p	2626	1932	1960	36
miR-263b-5p	2502	1719	1479	37
miR-1175-3p	1471	2416	2438	38
miR-12-5p	2413	2148	1577	39
miR-252b-5p	1896	2414	1999	40
miR-285-3p	2065	2368	1838	41
miR-3791-3p	43	346	2137	42

miR-9b-5p	67	364	2029	43
miR-190-5p	1372	1083	1694	44
miR-33-5p	1016	680	1587	45
miR-279e-3p	239	262	1534	46
miR-750-3p	529	677	1407	47
miR-13-3p	1347	728	197	48
miR-10-3p	1179	759	1022	49*)
miR-745-3p	231	1149	306	50
miR-252a-5p	1143	1132	307	51
miR-2944-3p	99	61	1081	52
miR-281-5p	781	960	934	53
miR-2a-1-5p	458	225	944	54
miR-283-5p	705	774	711	55
miR-750-5p	764	457	574	56
miR-276-5p	694	534	469	57*)
miR-92b-3p	477	250	653	58
miR-279b-5p	0	6	607	59*)
miR-277-3p	438	600	454	60
miR-7-5p	291	598	154	61
miR-965-5p	253	154	483	62
miR-133-5p	487	169	193	63*)
miR-279e-5p	1	21	402	64*)
miR-193-3p	293	380	389	65
miR-283-3p	366	199	275	66
miR-965-3p	291	356	175	67
miR-263a-3p	328	155	116	68*)
miR-252b-3p	207	162	319	69*)
miR-1175-5p	223	307	164	70*)
miR-71-3p	244	157	289	71*)
miR-210-3p	287	122	54	72
miR-100-3p	140	271	202	73*)
miR-137-3p	247	117	24	74
miR-305-3p	236	155	170	75*)
miR-275-5p	233	232	128	76*)
miR-993-5p	169	208	184	77*)
miR-998-3p	8	29	202	78
miR-282-5p	126	199	22	79
miR-278-3p	186	140	70	80
miR-iab-4-5p	177	79	63	81
miR-309-3p	4	27	174	82
miR-9b-3p	15	34	168	83*)
miR-1-5p	147	41	66	84*)
bantam-5p	132	274	113	85*)
miR-9a-3p	124	76	54	86*)
miR-190-3p	27	27	121	87*)
miR-184-5p	93	54	115	88*)

miR-2944-5p	5	75	104	89
miR-34-3p	68	103	16	90*)
miR-998-5p	0	2	98	91
miR-282-3p	94	76	31	92
miR-13-5p	88	58	39	93*)
miR-31-3p	86	52	13	94*)
miR-307-5p	19	13	73	95
miR-2b-5p	70	34	73	96*)
miR-92a-5p	6	4	66	97*)
let-7-3p	29	15	59	98*)
miR-279a-5p	34	21	54	99*)
miR-124-5p	53	24	31	100*)
miR-375-5p	8	11	47	101*)
miR-210-5p	43	13	9	102*)
miR-745-5p	12	41	6	103*)
miR-3791-5p	0	2	38	104*)
miR-87-1-5p	32	24	30	105*)
miR-263b-3p	30	13	3	106*)
miR-iab-8-5p	29	13	12	107
miR-281-3p	17	27	25	108*)
miR-981-3p	27	23	8	109
miR-279d-5p	6	6	18	110*)
miR-33-3p	11	11	11	111*)
miR-iab-4-3p	11	4	3	112*)
miR-278-5p	6	9	7	113*)
miR-92b-5p	1	0	7	114*)
miR-285-5p	0	6	1	115*)
miR-193-5p	4	2	5	116*)
miR-219-5p	3	2	5	117
miR-309-5p	0	0	5	118*)
miR-87-2-5p	4	0	0	119*)
miR-153-3p	4	2	1	120
miR-315-3p	2	0	3	121*)
miR-219-3p	2	0	3	122
miR-12-3p	3	0	3	123*)
miR-iab-8-3p	3	2	3	124*)
miR-981-5p	3	0	3	125*)
miR-307-3p	2	0	3	126*)
miR-277-5p	2	2	1	127*)
miR-153-5p	1	2	2	128
miR-137-5p	1	2	1	129*)
miR-7-3p	2	0	1	130*)
miR-252a-3p	1	0	0	131*)
miR-317-5p	1	0	0	132*)

Table S2. Mature miRNA from 5p and 3p arms of all 66 pre-miRNAs in order from highest to lowest normalized read counts. (*, putative mature passenger miRNA (counts 54)).

Supplementary1 Figure S1.

bantam

Secondary structure: ΔG = −35.3kcal/mol

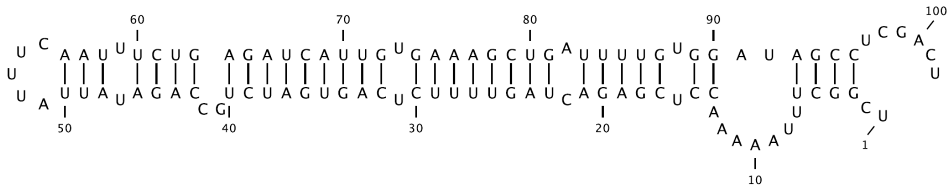

let-7

Secondary structure: ΔG = −88.5kcal/mol

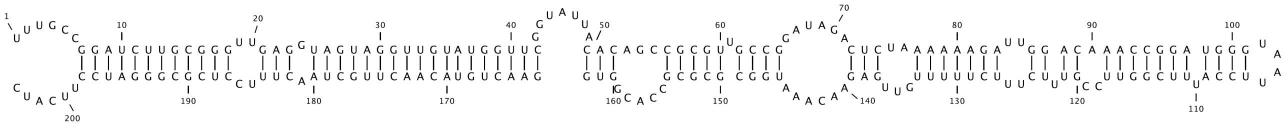

miR-1

Secondary structure: ΔG = −38.6kcal/mol

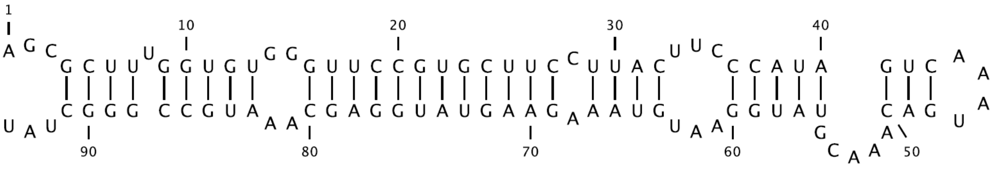

miR-2a-1

Secondary structure: ΔG = −39.5kcal/mol

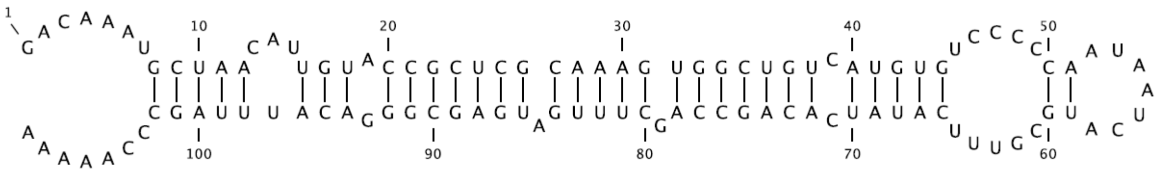

miR-2a-2

Secondary structure: ΔG = −41.8kcal/mol

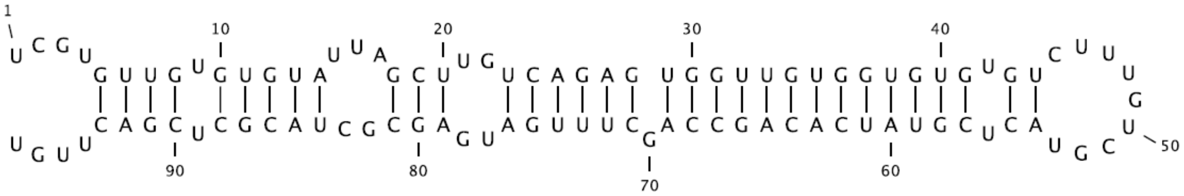

miR-2b

Secondary structure: ΔG = −38.5kcal/mol

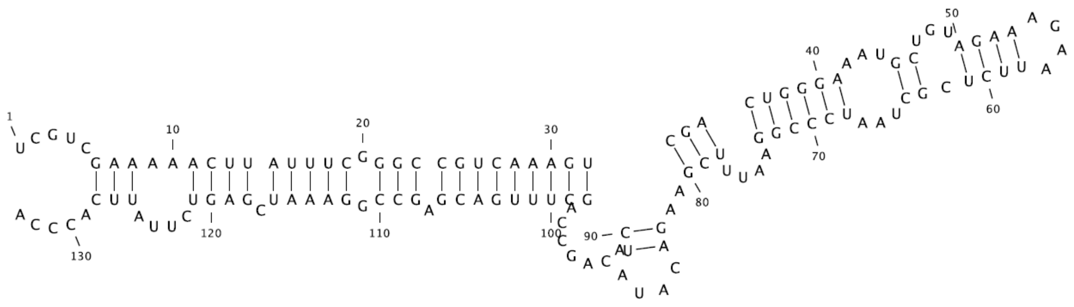

miR-7

Secondary structure: ΔG = −35.1kcal/mol

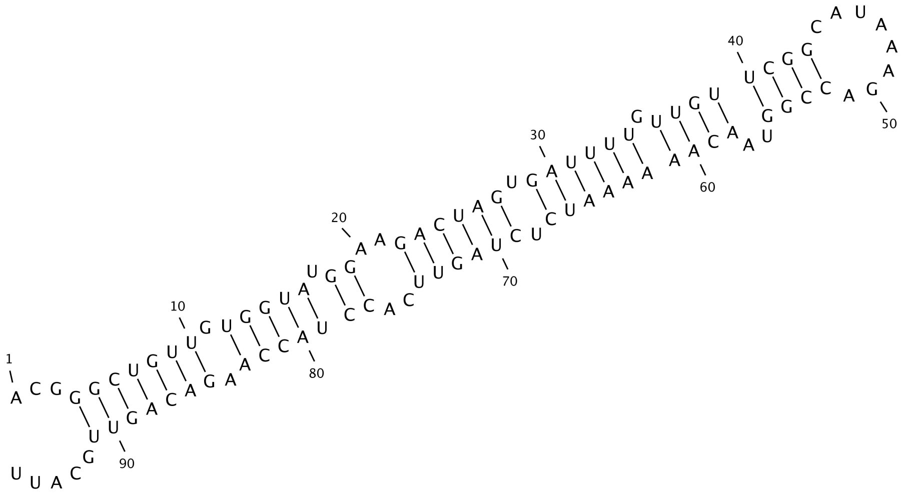

miR-8

Secondary structure: ΔG = −32.3kcal/mol

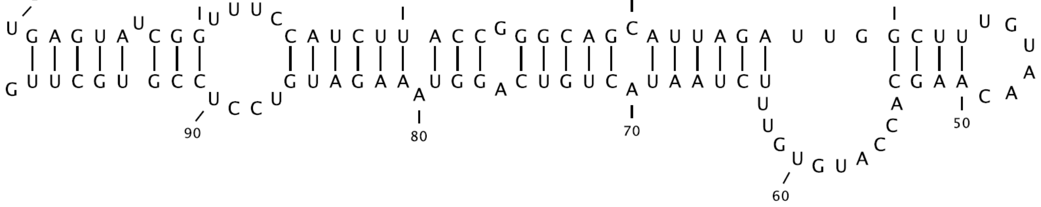

miR-9a

Secondary structure: ΔG = −36.0kcal/mol

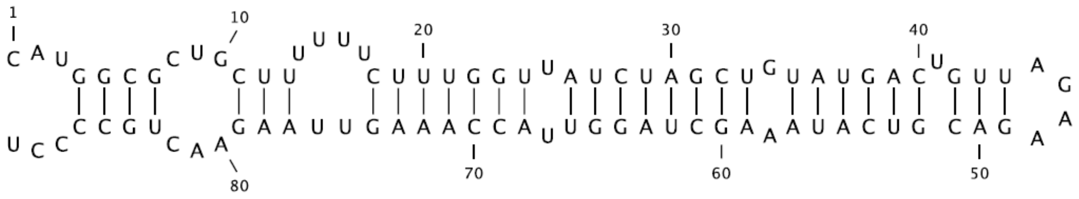

miR-9b

Secondary structure: ΔG = −38.5kcal/mol

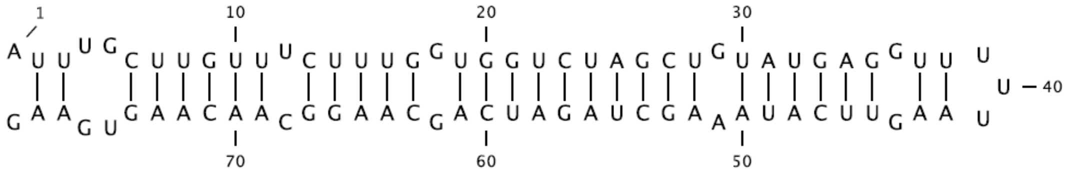

miR-10

Secondary structure: ΔG = −50.2kcal/mol

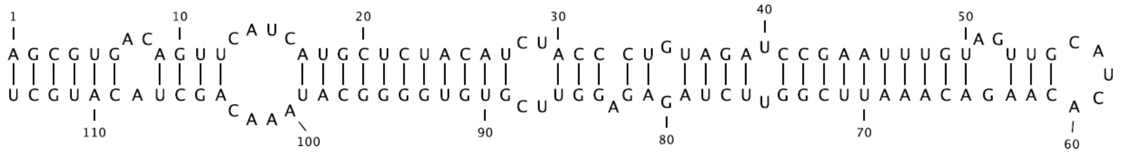

miR-12

Secondary structure: ΔG = −43.1kcal/mol

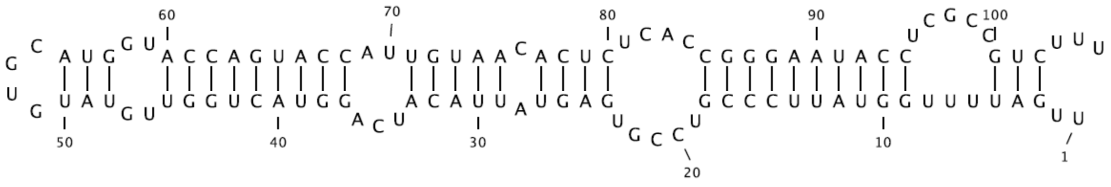

miR-13

Secondary structure: ΔG = −38.9kcal/mol

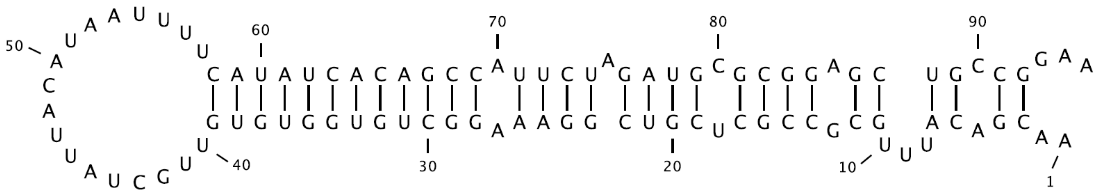

miR-31

Secondary structure: ΔG = −42.1kcal/mol

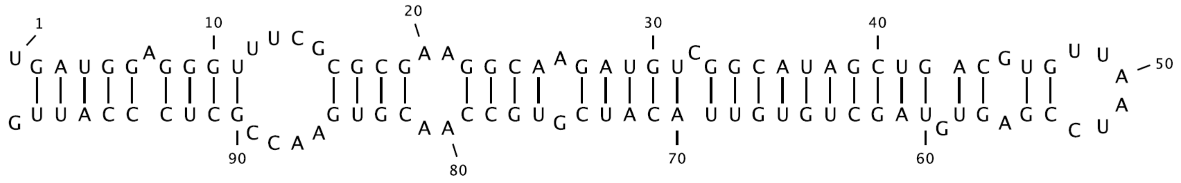

miR-33

Secondary structure: ΔG = −41.3kcal/mol

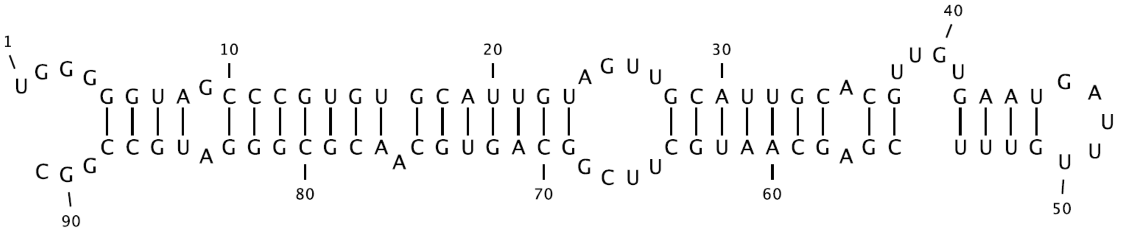

miR-34

Secondary structure: ΔG = −42.5kcal/mol

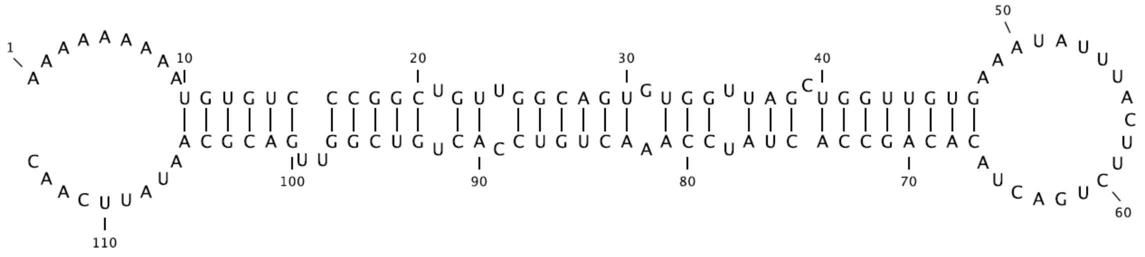

miR-71

Secondary structure: ΔG = −48.3kcal/mol

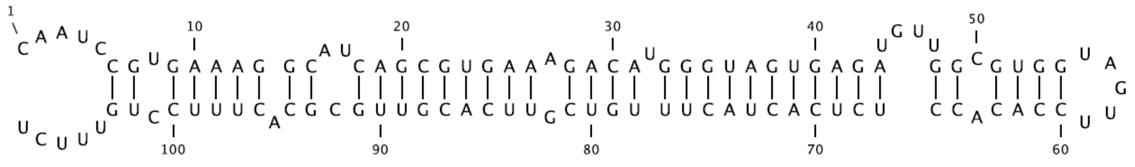

miR-87-1

Secondary structure: ΔG = −38.6kcal/mol

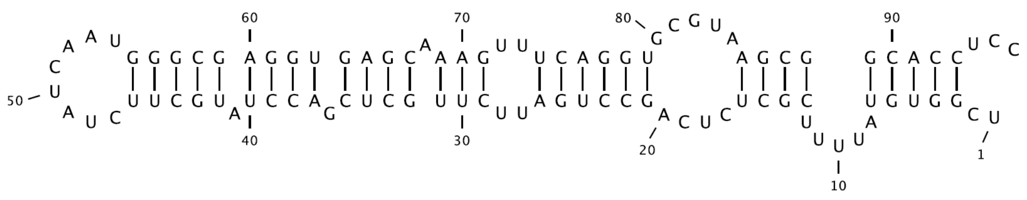

miR-87-2

Secondary structure: ΔG = −50.5kcal/mol

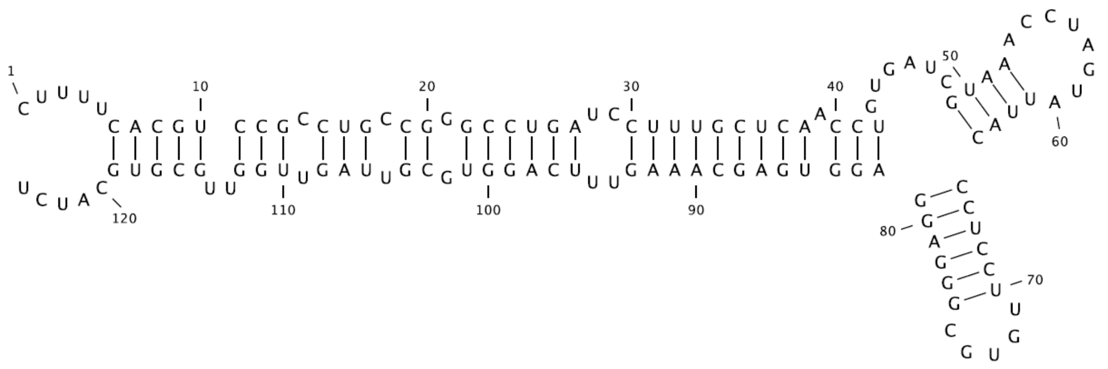

miR-92a

Secondary structure: ΔG = −38.9kcal/mol

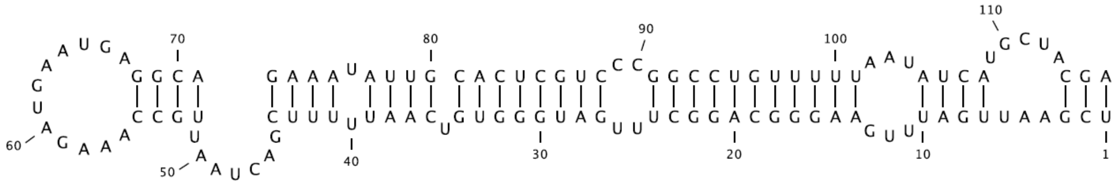

miR-92b

Secondary structure: ΔG = −21.5kcal/mol

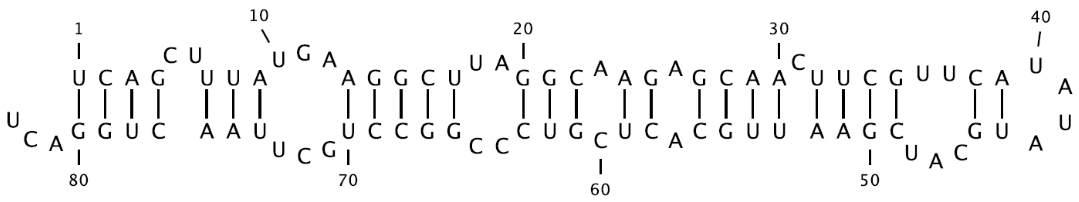

miR-100

Secondary structure: ΔG = −32.8kcal/mol

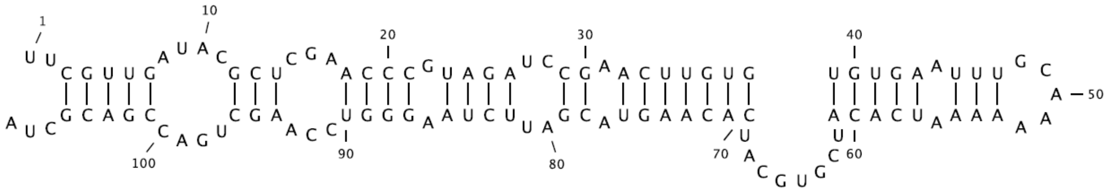

miR-124

Secondary structure: ΔG = −27.6kcal/mol

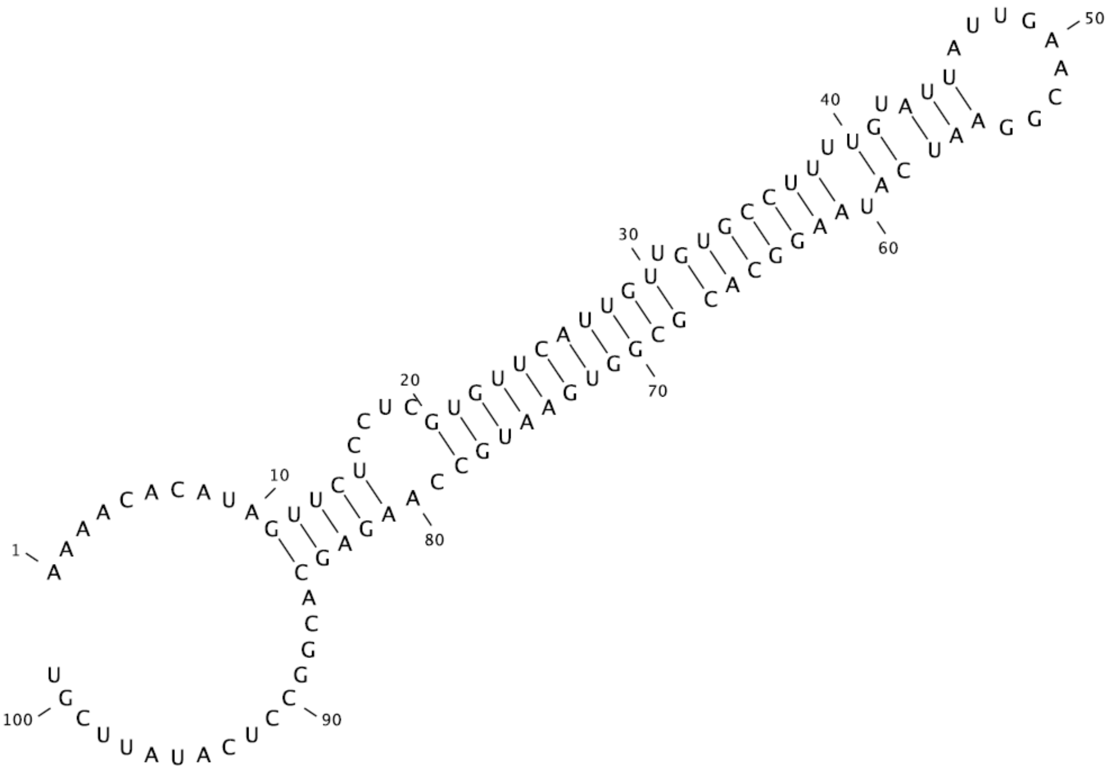

miR-125

Secondary structure: ΔG = −31.8kcal/mol

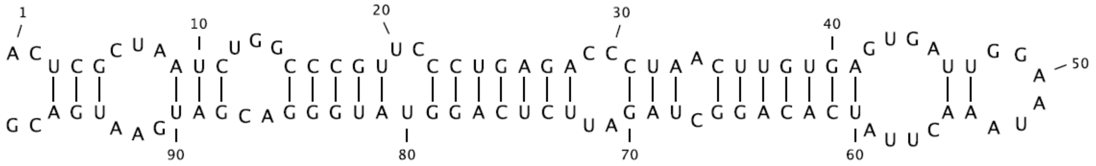

miR-133

Secondary structure: ΔG = −39.0kcal/mol

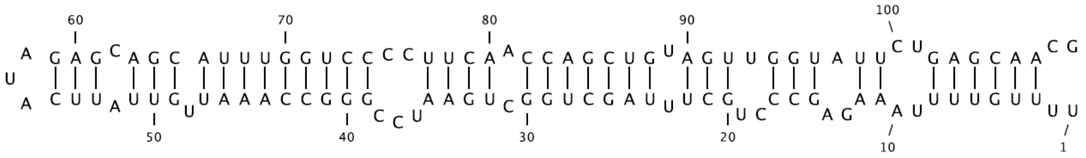

miR-137

Secondary structure: ΔG = −39.4kcal/mol

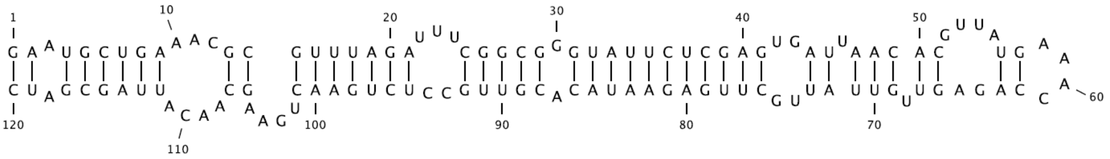

miR-153

Secondary structure: ΔG = −40.4kcal/mol

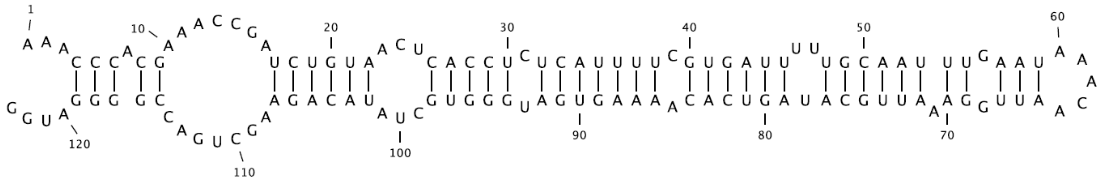

miR-184

Secondary structure: ΔG = −48.5kcal/mol

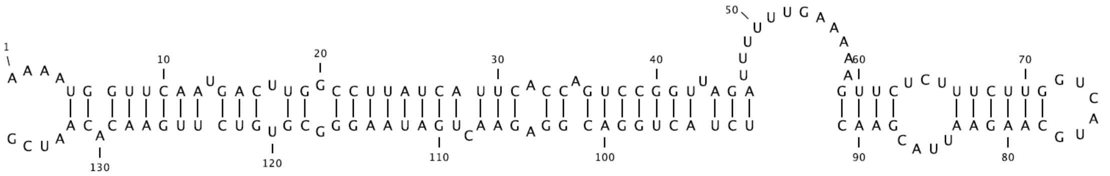

miR-190

Secondary structure: ΔG = −40.3kcal/mol

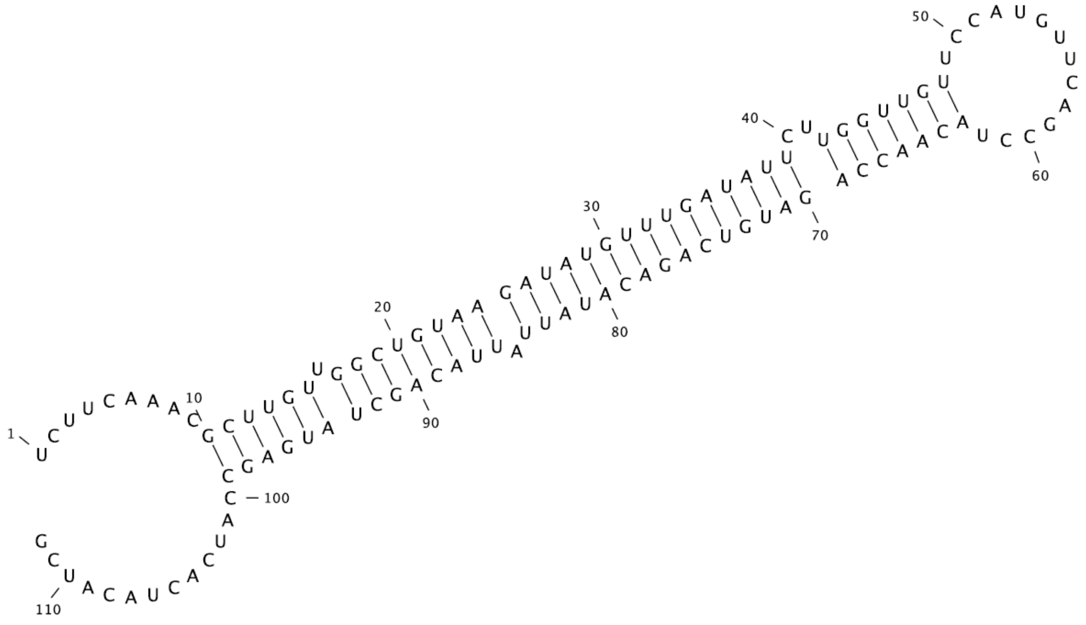

miR-193

Secondary structure: $\Delta G = -35.3$ kcal/mol

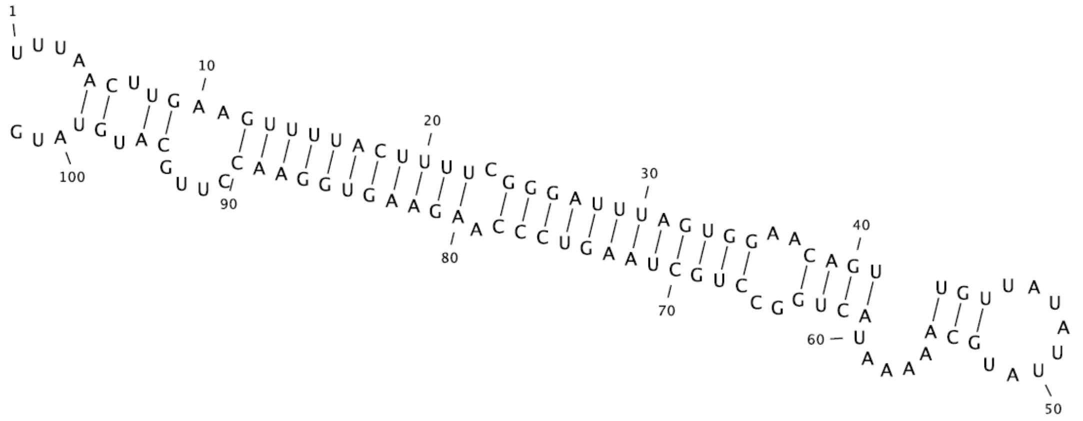

miR-210

Secondary structure: $\Delta G = -40.5$ kcal/mol

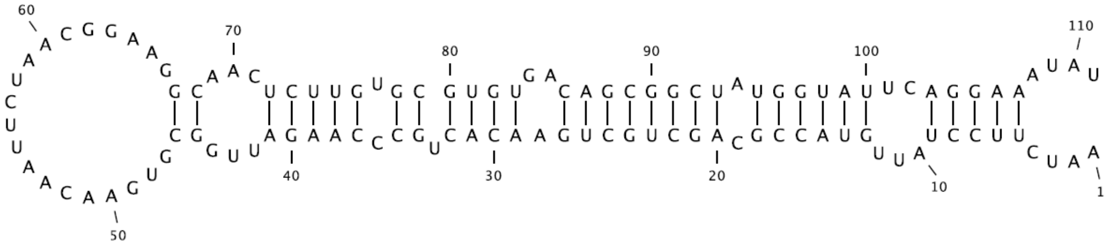

miR-219

Secondary structure: $\Delta G = -41.2$ kcal/mol

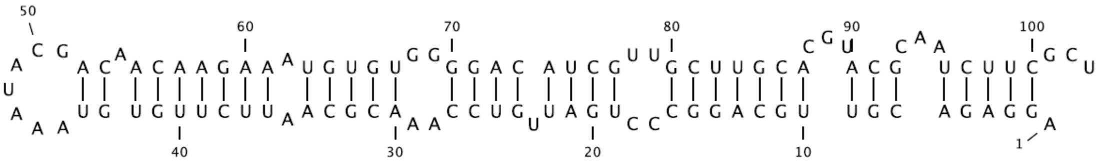

miR-252a

Secondary structure: $\Delta G = -31.0$ kcal/mol

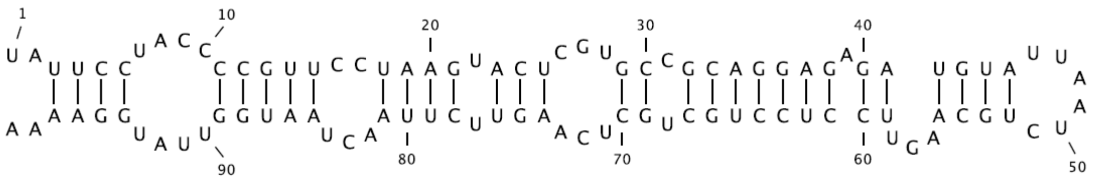

miR-252b

Secondary structure: ΔG = −39.0kcal/mol

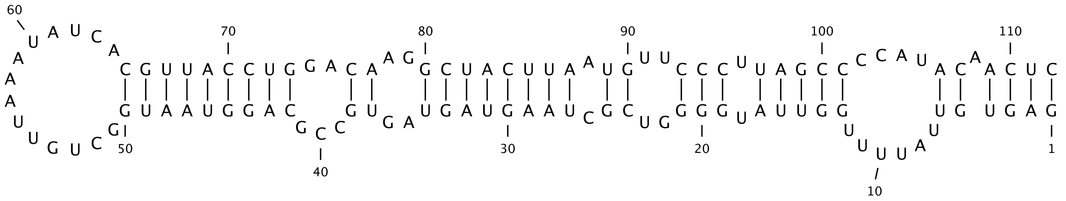

miR-263a

Secondary structure: ΔG = −84.2kcal/mol

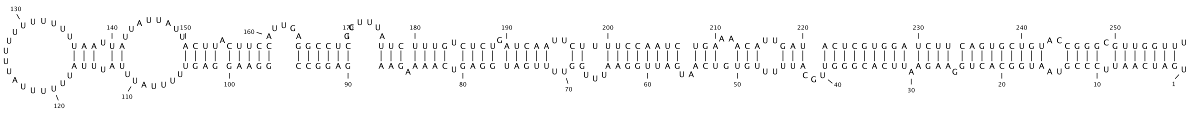

miR-263b

Secondary structure: ΔG = −33.9kcal/mol

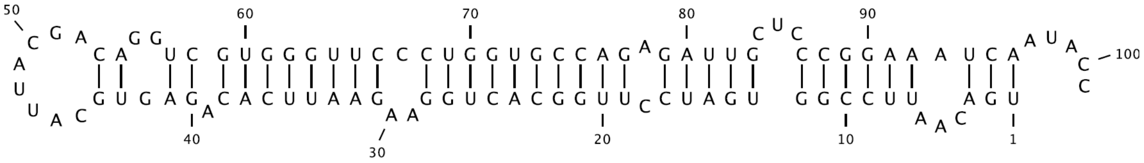

miR-275

Secondary structure: ΔG = −54.2kcal/mol

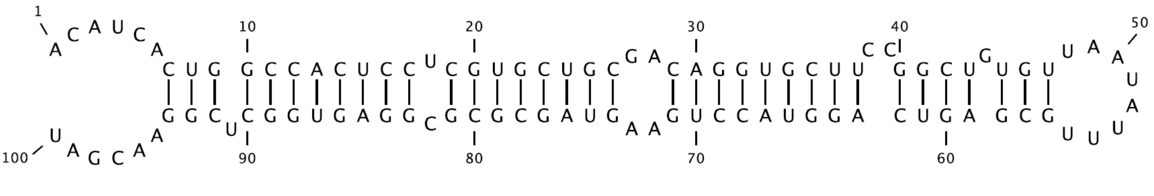

miR-276

Secondary structure: ΔG = −45.1kcal/mol

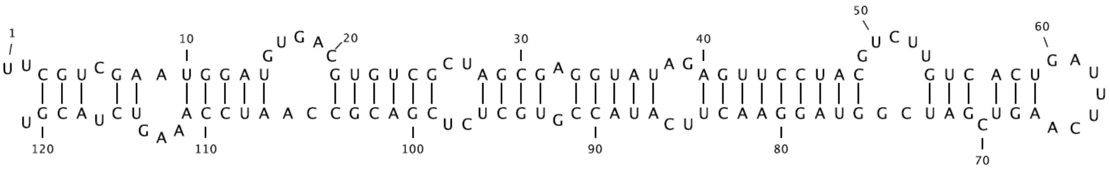

miR-277

Secondary structure: ΔG = −33.1kcal/mol

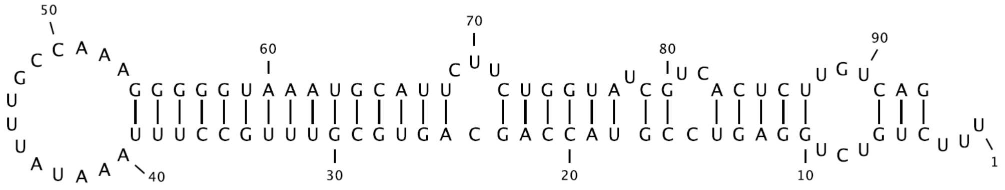

miR-278

Secondary structure: ΔG = −81.1kcal/mol

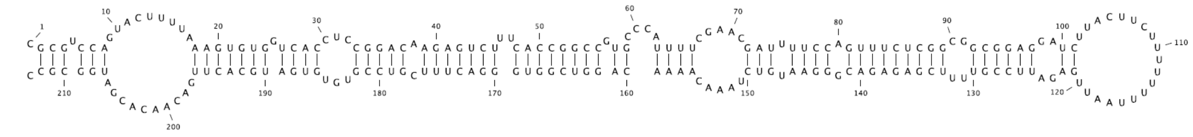

miR-279a

Secondary structure: ΔG = −38.0kcal/mol

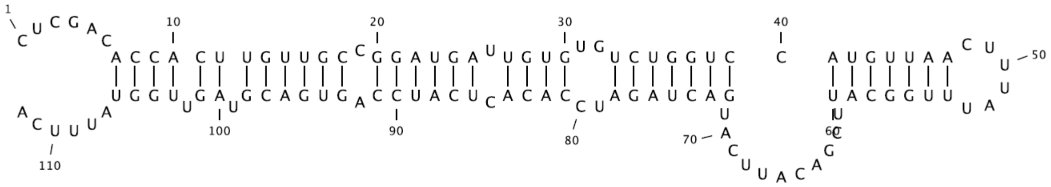

miR-279b

Secondary structure: ΔG = −37.0kcal/mol

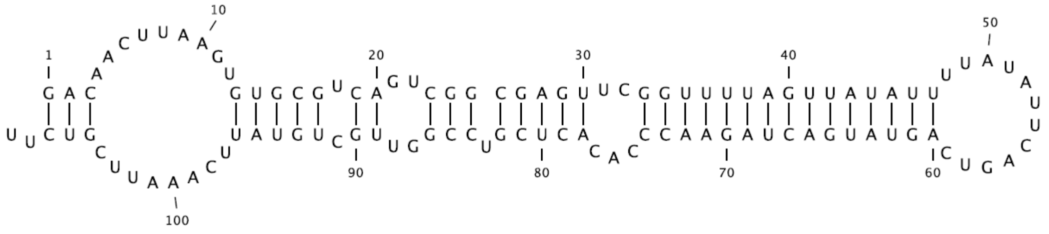

miR-279c

Secondary structure: ΔG = −39.7kcal/mol

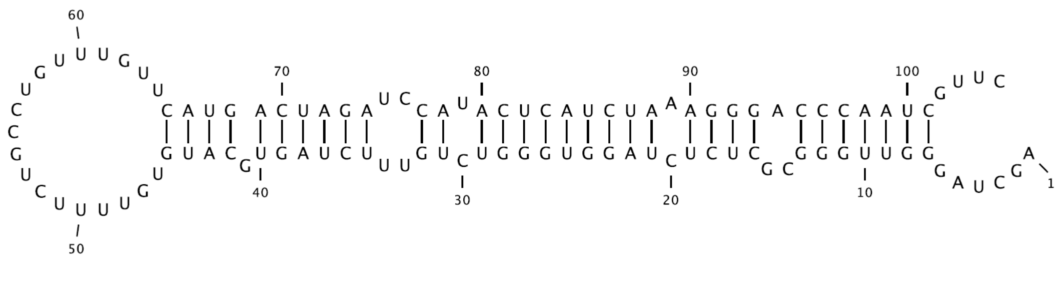

miR-279d

Secondary structure: ΔG = −42.8kcal/mol

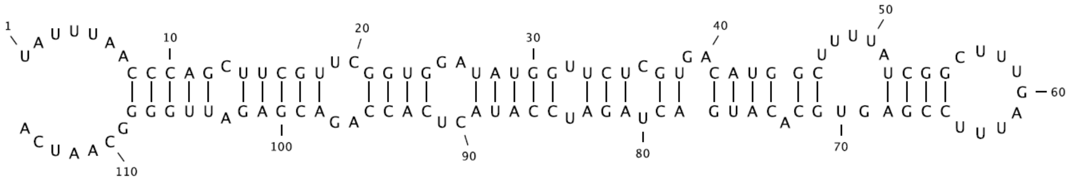

miR-279e

Secondary structure: ΔG = −40.8kcal/mol

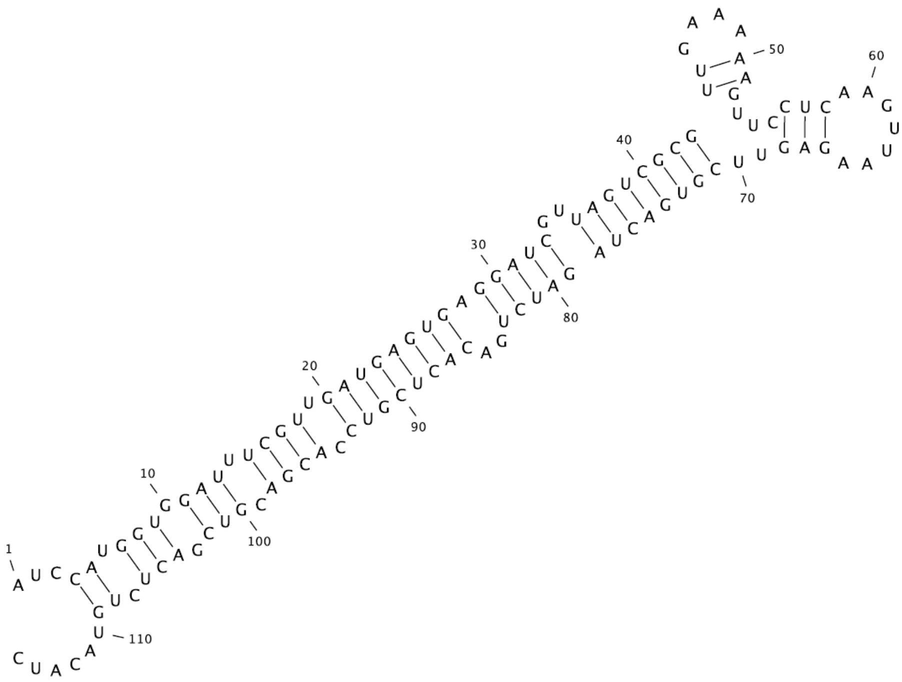

miR-281

Secondary structure: ΔG = −40.4kcal/mol

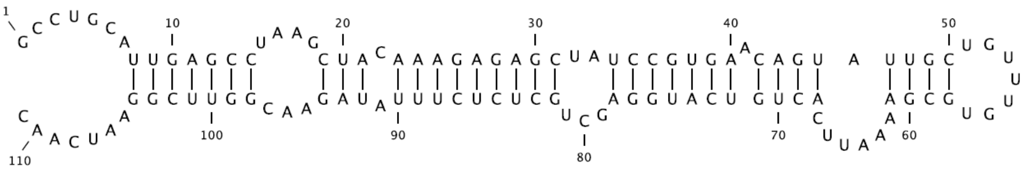

miR-282

Secondary structure: ΔG = −50.2kcal/mol

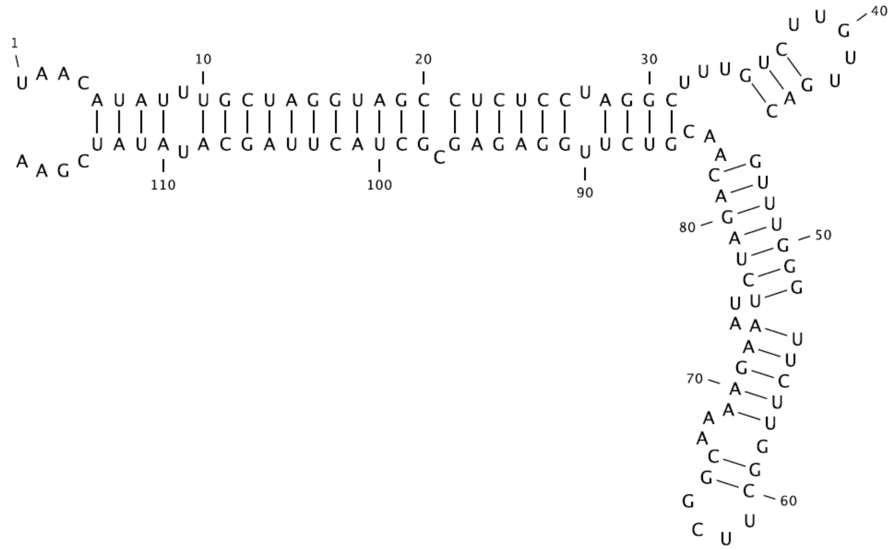

miR-283

Secondary structure: ΔG = −40.8kcal/mol

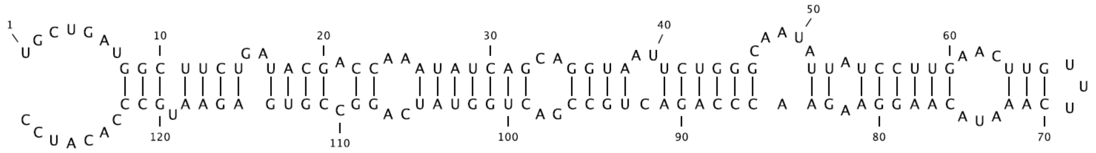

miR-285

Secondary structure: ΔG = −31.3kcal/mol

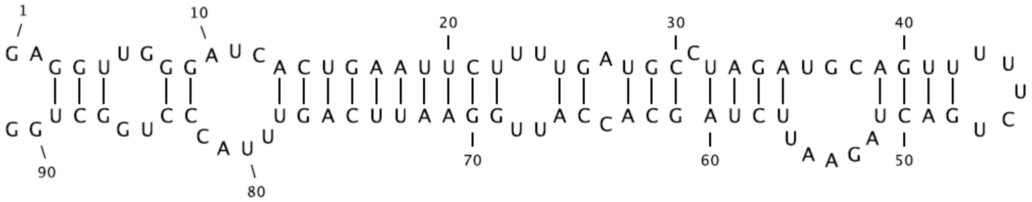

miR-305

Secondary structure: ΔG = –43.5kcal/mol

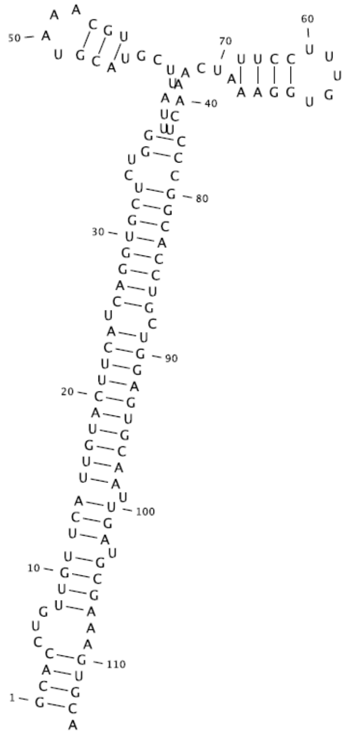

miR-307

Secondary structure: ΔG = –39.4kcal/mol

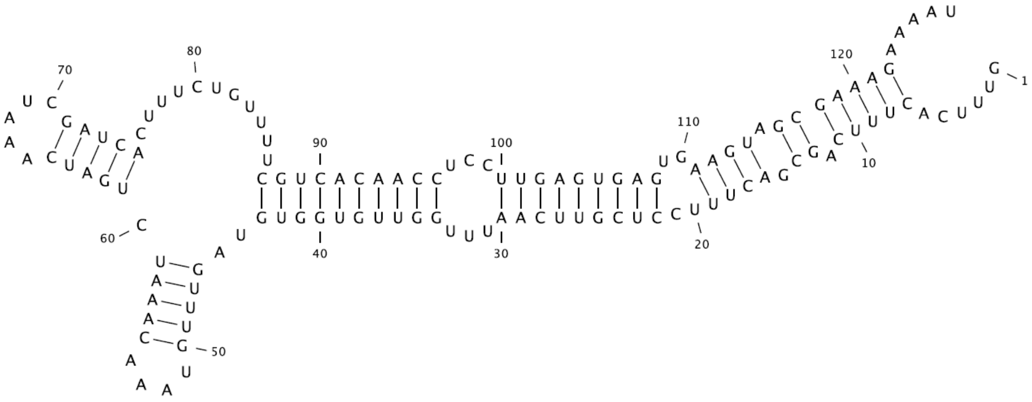

miR-309

Secondary structure: ΔG = −28.0kcal/mol

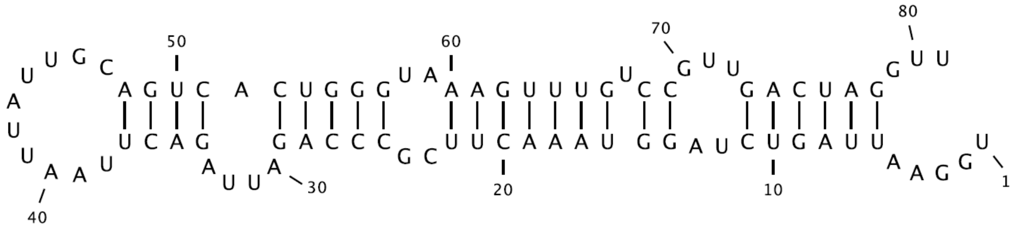

miR-315

Secondary structure: ΔG = −37.6kcal/mol

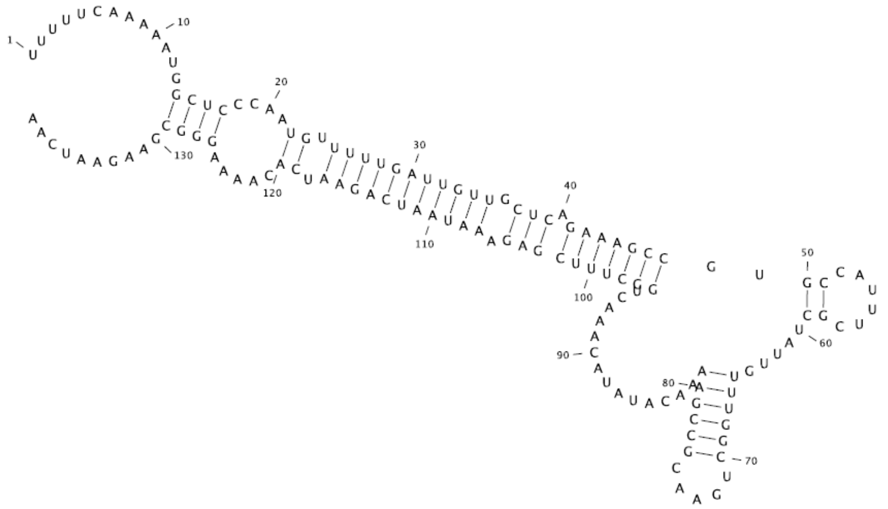

miR-317

Secondary structure: ΔG = −39.2kcal/mol

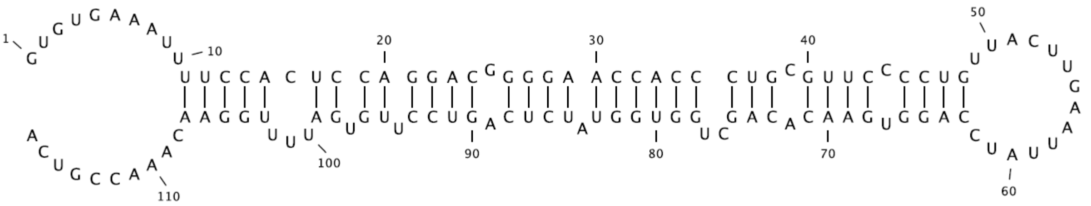

miR-375

Secondary structure: ΔG = −111.0kcal/mol

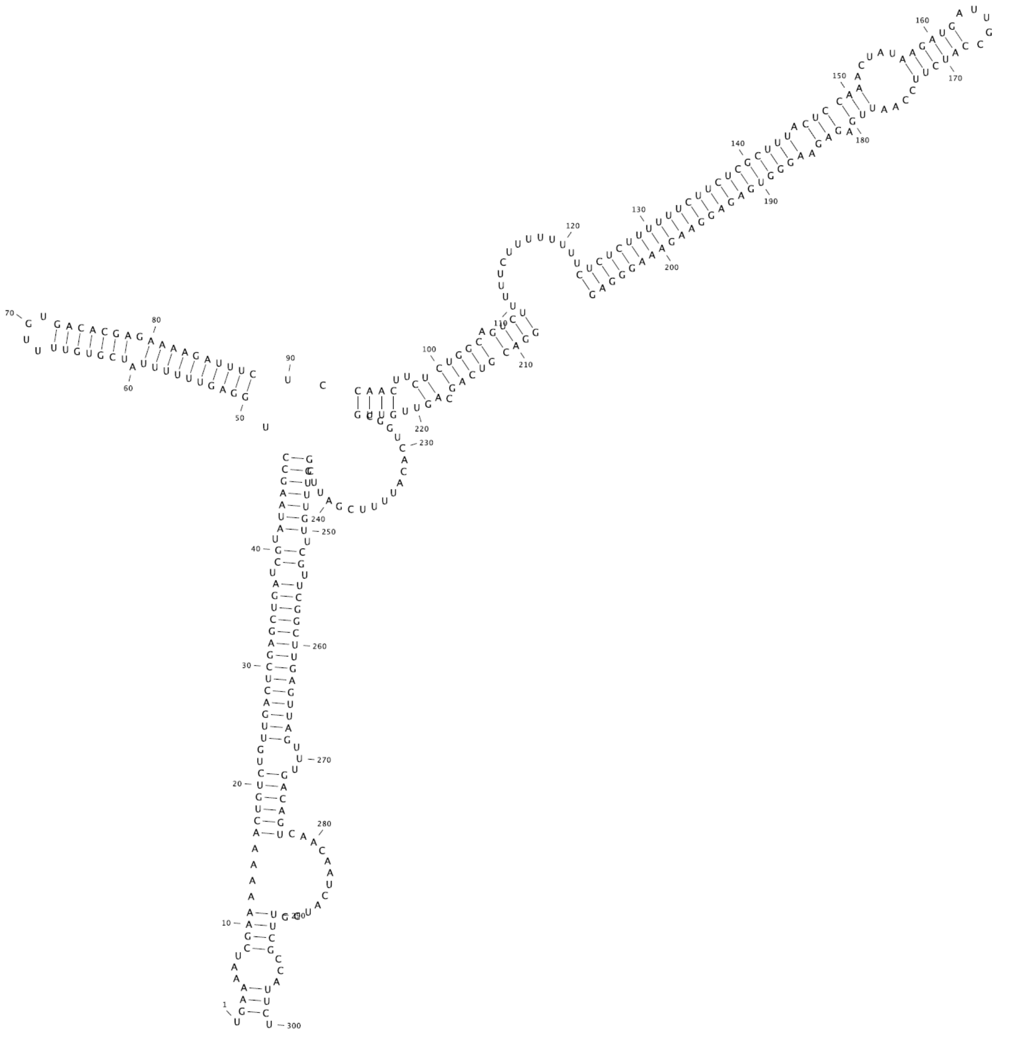

miR-745

Secondary structure: ΔG = −43.3kcal/mol

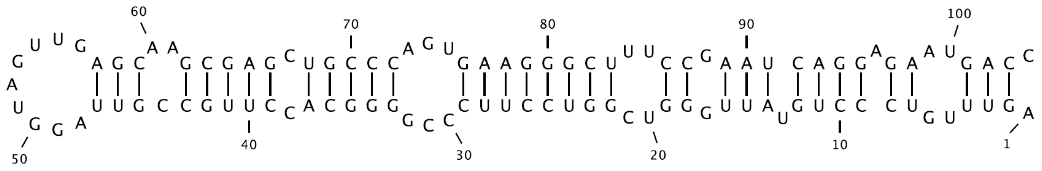

miR-750

Secondary structure: ΔG = −79.0kcal/mol

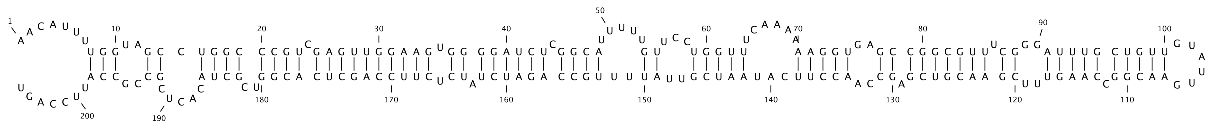

miR-965

Secondary structure: ΔG = −38.1kcal/mol

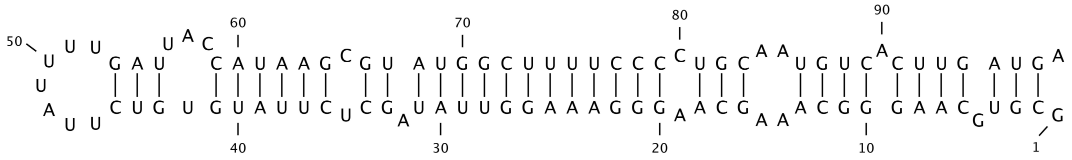

miR-981

Secondary structure: ΔG = −43.0kcal/mol

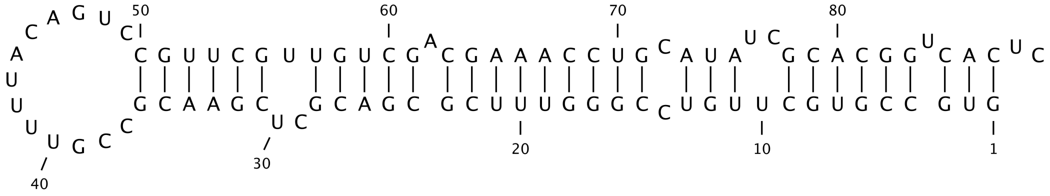

miR-993

Secondary structure: ΔG = −30.3kcal/mol

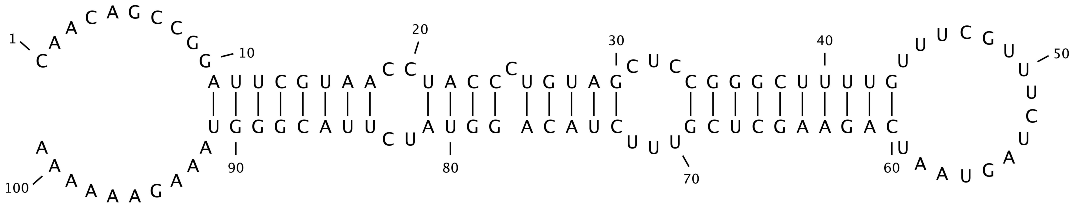

miR-998

Secondary structure: ΔG = −38.5kcal/mol

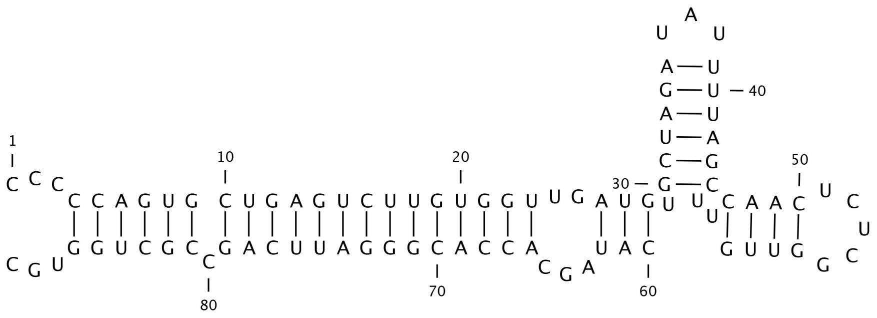

miR-1175

Secondary structure: ΔG = -37.8kcal/mol

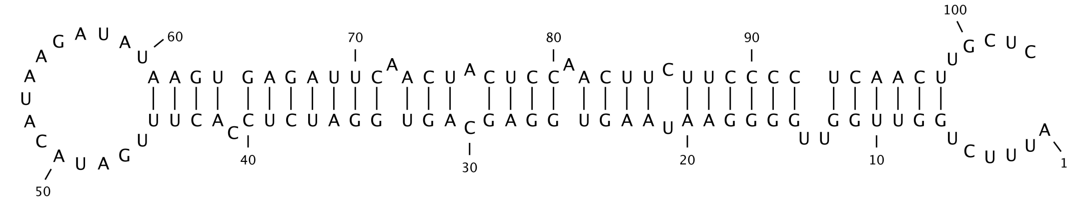

miR-2944

Secondary structure: ΔG = -34.0kcal/mol

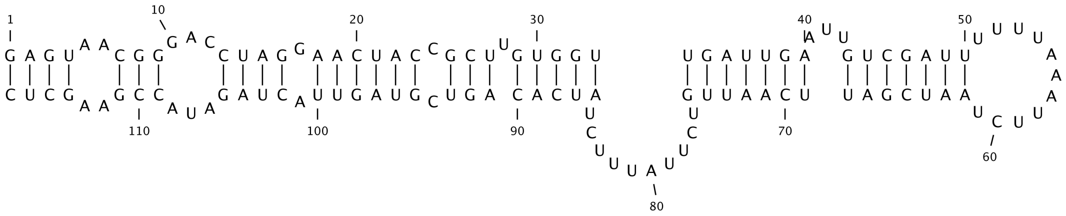

miR-3791

Secondary structure: ΔG = -28.8kcal/mol

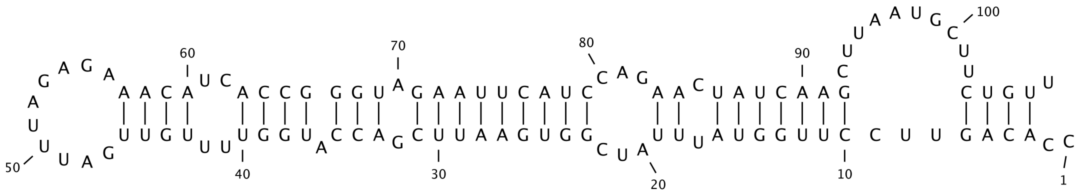

miR-iab-4

Secondary structure: ΔG = -32.0kcal/mol (mir-iab-4)

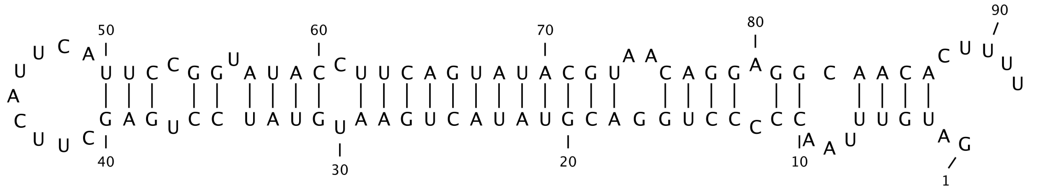

miR-iab-8

Secondary structure: ΔG = -46.9kcal/mol

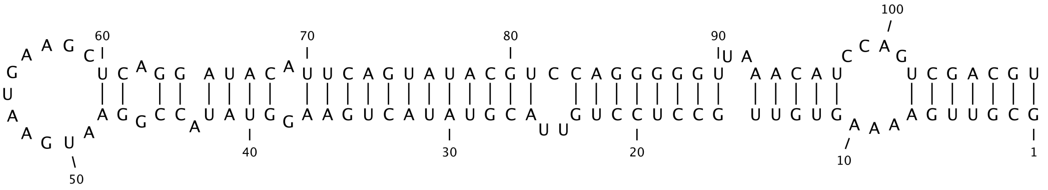

Figure S1. Secondary structure of conserved miRNA hairpins. The folding free energy (ΔG) is given for each predicted secondary structure of the miRNA hairpins.

Supplementary1 Table S3.

	Juvenile		Subadult		Adult	
	**5p[1)]**	**3p[2)]**	**5p[1)]**	**3p[2)]**	**5p[1)]**	**3p[2)]**
Nr miRNA with more than 10 reads in total	52	58	48	56	58	60
Nr mmiRNA with mmiRNA reads/total reads ≥50%	40	45	37	43	50	50
Nr 3′ template isomiRs with 3′ template isomiR reads/ total reads ≥21%	21	22	21	20	23	17
Nr 3′ non-template isomiRs with 3′ non-template isomiR reads/ total reads ≥4%	7	22	13	21	12	20
Nr of IsomiRs with SNP outside seed: SNP isomiR reads/total reads ≥2%	18	23	15	22	28	29
Nr 5′ template isomiRs with 5′ template isomiR reads/ total reads ≥4%	13	11	11	12	12	13
Nr 5′ non-template isomiRs with 5′ non-template isomiR reads /reads total ≥0.1%	14	22	8	15	11	17
Nr 5′ isomiR with SNP in seed: SNP in seed isomiR reads / total reads ≥3.5%	14	17	12	13	13	15

Table S3. Number (Nr) of conserved mature miRNA (mmiRNA) or isomiR at different percentage ratio of mmiRNA or isomiR read counts divided on total number of read counts for each miRNA arm. Only miRNAs with more than 10 reads (mmiRNA + isomiRs) are included.

1) Juvenile 5p: 14 miRNAs with less than 10 reads
Subadult 5p: 17 miRNAs with less than 10 reads
Adult 5p: 8 miRNAs with less than 10 reads

2) Juvenile 3p: 8 miRNAs with less than 10 reads
Subadult 3p: 10 miRNAs with less than 10 reads
Adult 3p: 6 miRNAs with less than 10 reads

Supplementary1 Table S4

miRNA	Juvenile		Subadult		Adult	
	H5p/L3p	H3p/L5p	H5p/L3p	H3p/L5p	H5p/L3p	H3p/L5p
miR-125	2220/1814<br>= 1.2		4376/996<br>= 4.4		3640/3032<br>= 1.2	
miR-2a-1		3234/458<br>= 7.1		1695/225<br>= 7.5	944/522<br>= 1.8	
miR-2a-2		3233/2283<br>=1.4		1711/1415<br>= 1.2	3700/524<br>= 7.0	
miR-282	126/94<br>= 1.3		199/76<br>= 2.6			31/23<br>= 1.3
miR-283	705/366<br>= 1.9		774/199<br>= 3.9		711/275<br>= 2.6	
miR-2944		99/5<br>=19,8	75/61<br>= 1.2			1081/104<br>= 10.4
miR-750	764/529<br>= 1.4			677/457<br>= 1.5		1407/574<br>= 2.5
miR-8	3067/2388<br>=1.3		3473/2349<br>=1.5		26504/12105<br>= 2.2	
miR-965		290/253<br>= 1.1		356/154<br>= 2.3	483/175<br>= 2.8	
miR-998	0	0		29/2<br>= 14.5		202/98<br>= 2.1
miR-219?	2,9/1.5<br>=1.9		0	0	4.6/3.2<br>= 1.4	
miR-153?		3,8/1,5<br>= 2.5	2,1/1,9<br>= 1.1		2,0/1,3<br>= 1.,5	

Table S4. Co-expression and arm switching of mature miRNA (normalized reads).H; most expressed arm; L, less expressed arm.

Supplementary1 Figure S2

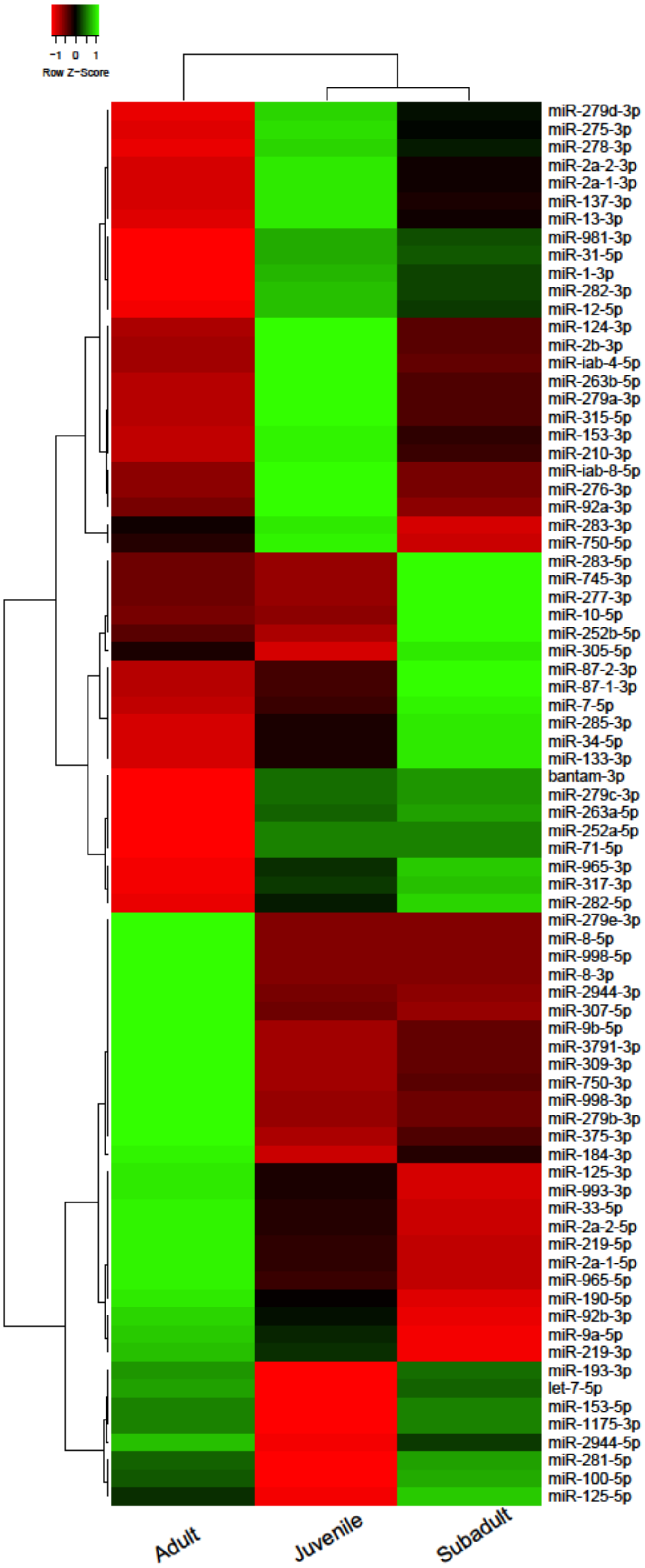

Figure S2. Heat map and clustering of mature guide miRNAs from juvenile, subadult and adult D. magna based on normalized read counts. Clustering of the three life stages is shown at the top of the heat map, while clustering of the miRNAs is shown to the left. The clustering was performed by using Pearson distance measurement and average linkage methods.

Supplementary1 Table S5.

miRNA	Mature miRNA sequence	Reads of mature miRNA			Length (nt)
		Juvenile	Subadult	Adult	
miR-nov-1-5p	GGUGAGCCGGCGUUUCGGGAUUU	9	6	1	23
miR-nov-1-3p	GUUUCGAACGUCGAGCCAACC	43	40	3	21
miR-nov-3-5p	UCUUGGUUGCUCGGUCUUUAGG	21	79	829	22
miR-nov-3-3p	UAAAGCUCGGCUAGCAGGAUCC	20	356	3017	22
miR-nov-4-5p	CCAGUUUAACAUAGCCCACAGA	1	0	9	22
miR-nov-4-3p	UCUGGGUUAUGAUUAAGACUGGG	181	847	4718	23
miR-nov-10-5p	UGAAGCAGAGGACUGCUUUGA	19	73	62	21
miR-nov-10-3p	UGAGAGCAGUUCUCUGCUUCAUU	0	2	3	23
miR-nov-12-5p	GGGGGGAACUUUACUCAGUUUGAU	1	2	0	24
miR-nov-12-3p	UCACUGGGUACGUUCGCCCUUG	2	17	60	22
miR-nov-2-5p		nd	nd	nd	
miR-nov-2-3p	CACUGGGUAUUCAUUGUAGGGUCGA	18	1042	4468	25
miR-nov-5-5p	GUGAGCUUGGAUUUAAAAGAA	1	0	1	21
miR-nov-5-3p	CUUUUUUUUCUCCACUCACAG	30	15	29	21
miR-nov-6-5p	AAUGUGAUAUUUUAUCCUUCUA	24	40	54	22
miR-nov-6-3p	UAGAAGGAUAAAAUAUCACAUU	24	42	53	22
miR-nov-7-5p		nd	nd	nd	
miR-nov-7-3p	UUGCACUAACCGGUAGUGGGACG	20	243	248	23
miR-nov-8-5p		nd	nd	nd	
miR-nov-8-3p	UUGCACUGGCCUGCCCAGGGGCG	0	17	10	23
miR-nov-9-5p		nd	nd	nd	
miR-nov-9-3p	UUGCACUGUACGGUUCGAGGCGA	827	5793	5007	23
miR-nov-11-5p	UUUAAAAUCUGACUGAGAGGUU	4	32	125	22
miR-nov-11-3p		nd	nd	nd	
miR-nov-13-5p	AAAUCUCAUACGGUAAUUUGGA	100	105	89	22
miR-nov-13-3p		nd	nd	nd	

Table S5. Novel miRNAs in *D. magna* (normalized read counts. nt: nucleotides. nd: not detected.

Supplementary1 Figure S3.

miR-nov-2

Secondary structure: ΔG = -25.8kcal/mol

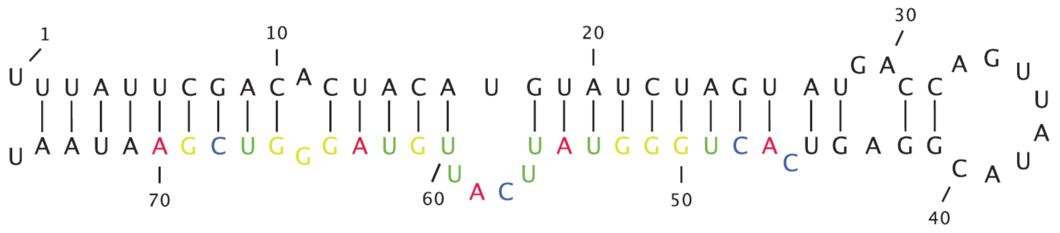

miR-nov-5

Secondary structure: ΔG = -18.4kcal/mol

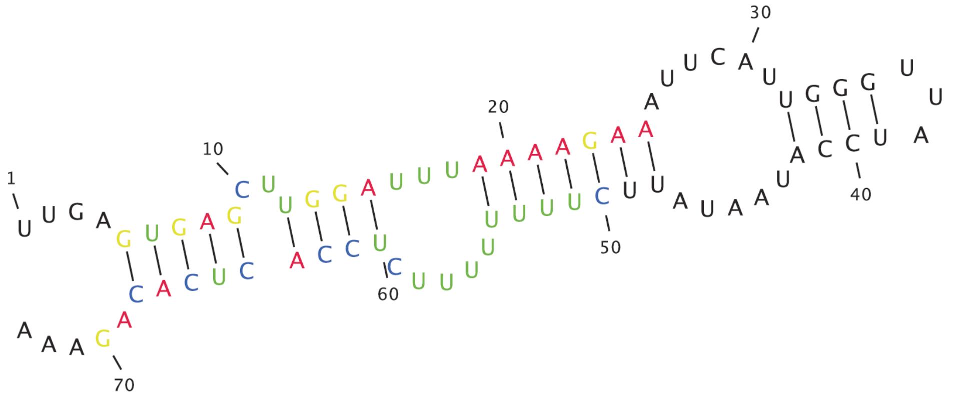

miR-nov-6

Secondary structure: ΔG = -48.6kcal/mol

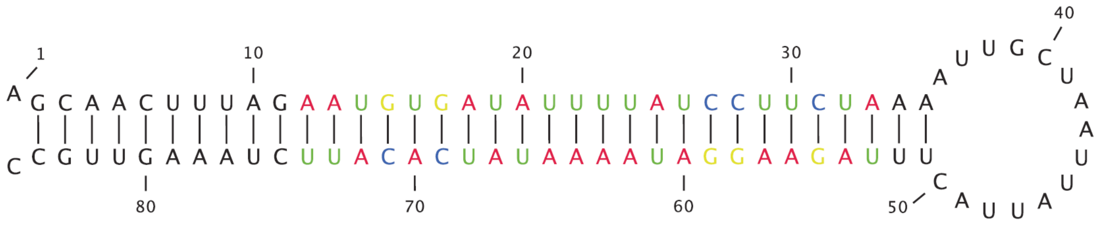

miR-nov-7

Secondary structure: ΔG = -31.1kcal/mol

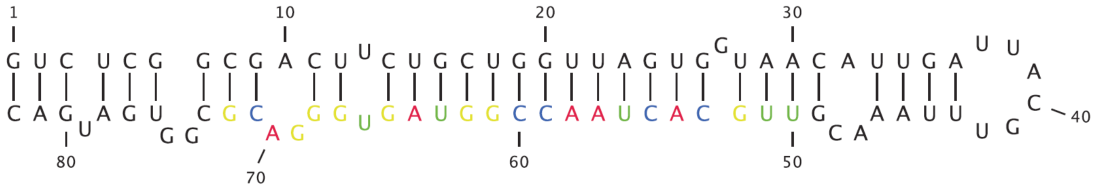

miR-nov-8

Secondary structure: ΔG = -27.0kcal/mol

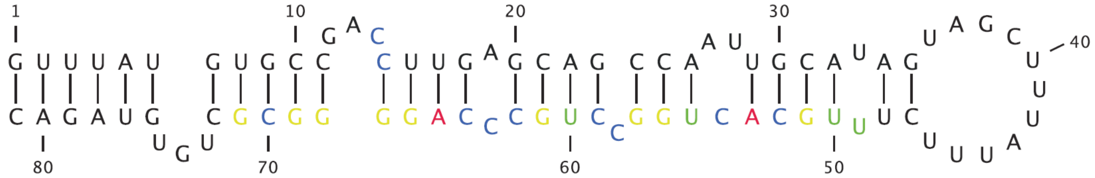

miR-nov-9

Secondary structure: ΔG = -30.5kcal/mol

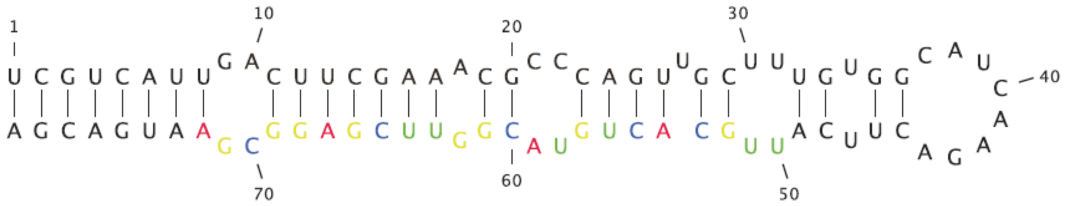

miR-nov-11

Secondary structure: ΔG = -34.1kcal/mol

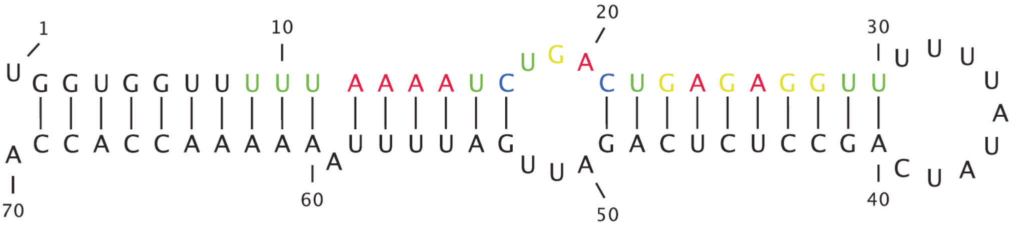

miR-nov-13

Secondary structure: ΔG = -63.1kcal/mol

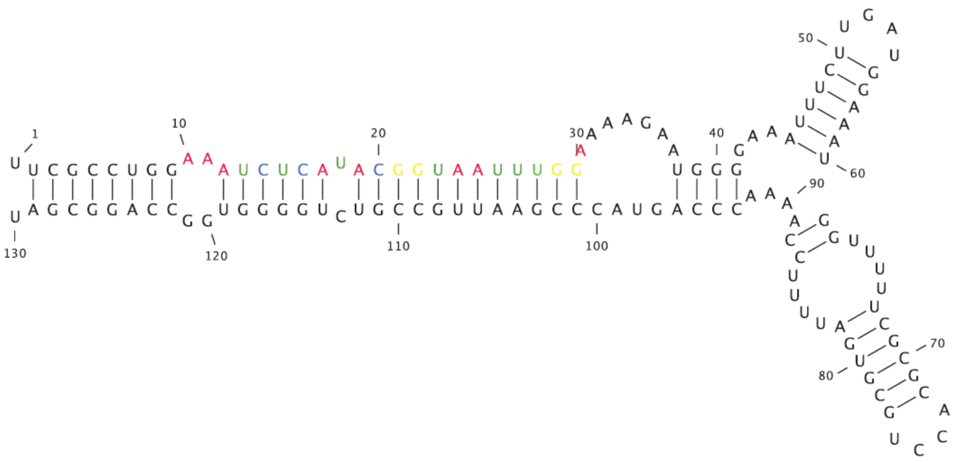

Figure S3. Secondary structure of hairpins of putative novel miRNAs.The folding free energy (ΔG) is given for each predicted secondary structure of the miRNA hairpins.

Supplementary1 Table S6.

	Genomic localization *D. magna*			Genomic localization *D. pulex*[2)]			Notes
	Scafold	Strand	pre-miR coordinates on + strand	Scafold	Strand	pre-miR coordinates on + strand	
miR-nov-1	243	-	73895-73959				
miR-nov-3	548	-	2085-2145	24	-	361693-361754	
miR-nov-4	548	-	1700-1757	24	-	361301-361359	
miR-nov-10	1036	-	421101-421163	43	-	318393-318465	Based on 5p miRNA (3p aligned to sc 111 (probably not miRNA in *D. pulex)*)
miR-nov-12	2190	-	1745310-1745371	32	-	69152-69214	
miR-nov-2	548	-	2233-(2295)[1)]	24	-	361858-361920	
miR-nov-5	642	-	169265-169330	35	+	749346-749414	Uncertain miRNA in *D.pulex*
miR-nov-6	781	-	364257-364319			nd	
miR-nov-7	1036	-	1020892-(10209549)[1)]	13	+	1739741-1739803	
miR-nov-8	1036	-	1020730-(1020791)[1)]	13	+	1739872-1739933	
miR-nov-9	1036	-	1020540-(1020602)[1)]	13	+	1740035-1740097	
miR-nov-11	1036	-	(420885)[1)]-420940	16	-	921275-921315	Probably not miRNA in *D. pulex*
miR-nov-13	2703	-	2034-2147	1	-	1848248-1848369	

Table S6. Genomic localization of putative novel miRNAs in *D. magna* and *D. pulex*.
[1)] Coordinates in parenthesis are putative miRNA sequence ends. [2)] Putative coordinates are based on pre-miRNA sequences from *D. magna*. nd, not detected.

Supplementary1 Table S7.

	Cluster in *D. magna*			Clusters in *D. pulex*[2)]		
**miRNA**	**Scaffold**	**Strand**	**pre-miR coordinates of + strand (Scaffold)**	**Scaffold**	**Strand**	**pre-miR coordinates of + strand (Scaffold)**
miR-71	24	-	3280380-3280445	80	+	240430-240495
miR-2b	24	-	3280225-3280310	80	+	240558-240644
miR-13	24	-	3280063-3280125	80	+	240731-240792
miR-2a-1	24	-	3279939-3280007	80	+	240864-240934
miR-2a-2	24	-	3279749-3279812	80	+	241044-241107
miR-750	243	-	73854-74005	113	+	97296-97448
miR-nov-1	243	-	73895-73959	113	+	*)
miR-1175	243	-	73661-73726	113	+	97594-97659
miR-100	243	+	423404-423480	71	-	446641-446720
let-7	243	+	423613-423780	71	-	446343-446521
miR-125	243	+	424677-424739	71	-	445364-445427
miR-nov-2	548	-	2233-(2295)[1)]	24	-	361858-361920
miR-nov-3	548	-	2085-2145	24	-	361693-361754
miR-309	548	-	1861-1920	24	-	361463-361522
miR-nov-4	548	-	1700-1757	24	-	361301-361359
miR-nov-7	1036	-	1020892-1020949)[1)]	13	+	1739741-1739803
miR-nov-8	1036	-	1020730-1020791)[1)]	13	+	1739872-1739933
miR-nov-9	1036	-	1020540-1020602)[1)]	13	+	1740035-1740097
miR-87-2	1067	-	2294-2379	1	+	2190896-2190980
miR-87-1	1067	-	2113-2176	1	+	2191079-2191142
miR-275	1361	-	9029-9093	4	+	1790740-1790804
miR-305	1361	-	8573-8660	4	+	1791164-1791251
miR-317	1361	+	859057-859121	4	-	1243963-1244027
miR-277	1361	+	860062-860128	4	-	1242975-1243041
miR-34	1361	+	861121-861188	4	-	1242049-1242114
miR-263a	1361	-	2445783-2446013	87	+	475620-475711
miR-263b	1361	-	2445501-2445563	87	+	475817-475879
miR-279e	2190	-	1745878-1745953	32	-	69703-69769
miR-9b	2190	-	1745746-1745804	32	-	69575-69633
miR-2944	2190	-	1745587-1745679	32	-	69416-69503
miR-998	2190	-	1745456-1745530	32	-	69280-69354
miR-nov-12	2190	-	1745310-1745371	32	-	69152-69214
miR-279b	2190	-	1744801-1744864	32	-	68663-68726
miR-3791	2190	-	1744660-1744720	32	-	68520-68579
miR-279d	2385	-	1033657-1033733	43	+	177095-177172
miR-279c	2385	-	1033185-1033249	43	+	177501-177566
miR-283	1551	-	399-484	1	-	1848726-1848812
miR-nov-13	2703	-	2032-2147	1	-	1848253-1848369
miR-12	2703	-	1623-1683	1	-	1847845-1847905
miR-92a	2957	+	30615-30692	38	+	876143-876215
miR-92b	2957	+	30791-30850	38	+	876332-876391

Table S7. miRNA clusters within 10 000 bps in the *D. magna* and *D. pulex* genomes.
[1)] Coordinates in parenthesis are putative pre-miRNA sequence ends. [2)] Putative coordinates are based on pre-miRNA sequences from *D. magna*. *) A possible pre-miRNA that fold to ΔG= -26,6 kcal/mole, but with different seed sequences compared to miR-nov-1 in the pre-miR-750 sequence of *D. magna*.)

Supplementary1 Table S8.

	Juvenile (JU)		Subadult (SA)		Adult (AD)		Mean JU+SA+AD		
	**5p[1)]**	**3p[2)]**	**5p[1)]**	**3p[2)]**	**5p[1)]**	**3p[2)]**	**5p+3p**	**5p**	**3p**
Mean mature miRNA reads of total reads (%)	63.5%	65.6%	65.6%	63.3%	68.2%	67.7%	65.7%	65.8%	65.3%
Mean 3' template isomiRs reads of total reads	24.3%	19.2%	21.9%	20.1%	21.1%	17.4%	20.7%	22.4%	18.9%
Mean 3' non-template isomiRs reads of total reads	2.0%	4.8%	2.7%	5.9%	2.6%	4.0%	3.7%	2.4%	4.9%
Mean isomiRs reads with SNP outside seed of total reads	2.1%	2.2%	2.0%	2.0%	2.7%	2.2%	2.2%	2.3%	2.1%
Mean percentage sum of isomiRs with intact seed of total reads	*28.4%*	*26.3%*	*26.5%*	*28.0%*	*26.4%*	*23.6%*	*26.5%*	*27.1%*	*25.9%*
Mean 5' template isomiRs reads of total reads	4.0%	4.5%	3.6%	5.5%	2.4%	5.1%	4.2%	3.3%	5.0%
Mean 5' non template isomiRs reads of total readsal	0.06%	0.07%	0.13%	0.09%	0.06%	0.05%	0.08%	0.08%	0.07%
Mean isomiRs reads with SNP in seed of total reads	4.0%	3.7%	4.2%	3.1%	2.9%	3.2%	3.5%	3.7%	3.5%
Mean percentage sum of isomiRs with "new" seed of total reads	*8.1%*	*8.3%*	*7.9%*	*8.7%*	*5.4%*	*8.4%*	*7.8%*	*7.1%*	*8.5%*

Table S8. The average percentage ratio of the sum of read counts from all conserved mature miRNAs and all the different isomiR groups each divided by total read counts. Only miRNAs with more than 10 reads (mature miRNA + isomiRs) are included.

[1)] JU 5p: 14 miRNAs with less than 10 reads
SA 5p: 17 miRNAs with less than 10 reads
AD 5p: 8 miRNAs with less than 10 reads

[2)] JU 3p: 8 miRNAs with less than 10 reads
SA 3p: 10 miRNAs with less than 10 reads
AD 3p: 6 miRNAs with less than 10 reads

Supplementary1 Figure S4.

a)

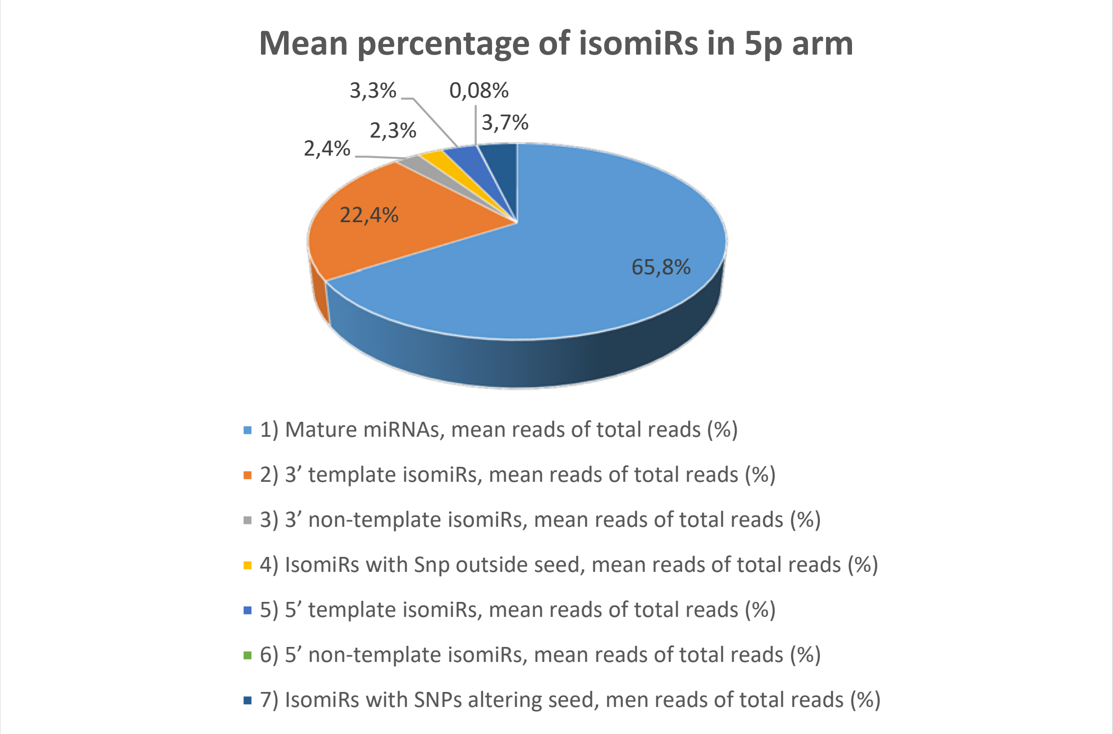

b)

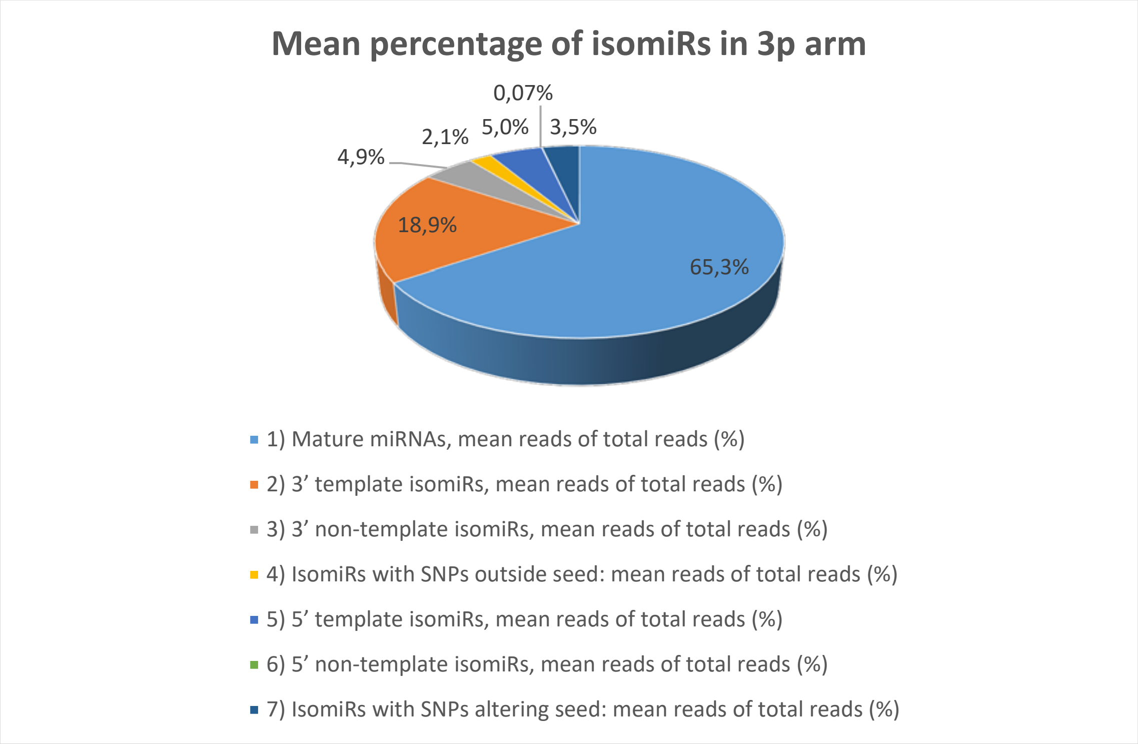

Figure S4. Mean percentage ratio of mature miRNA and isomiR reads out of total reads for the 5p arm (a) and the 3p arm (b).
