# Supplementary material for: MicroRNAs in Daphnia magna identified and characterized by deep sequencing, genome mapping and manual curation
Source: Sci Rep. 2019 Nov 4;9:15945. doi: 10.1038/s41598-019-52387-z (PMC6828783; doi:10.1038/s41598-019-52387-z)
Supplement: Supplementary file 3 — Supplementary3 information [file 41598_2019_52387_MOESM3_ESM.docx]

# MicroRNAs in *Daphnia magna* identified and characterized by deep sequencing, genome mapping and manual curation.

Dag H. Coucheron^1*)^, Marcin W. Wojewodzic^2,3*)^ & Thomas Bøhn^4*)^

^1)^ MIRG, Department of Medical Biology, UiT The Arctic University of Norway, N-9037 Tromsø, Norway

E-mail: [dag.coucheron@uit.no](mailto:dag.coucheron@uit.no)

Tel. +47 77644657

^2)^ School of Biosciences, University of Birmingham, Edgbaston, Birmingham, B15 2TT UK

E-mail: [M.Wojewodzic@bham.ac.uk](mailto:M.Wojewodzic@bham.ac.uk)

^3)^ Research Department, Cancer Registry of Norway, Ullernchausseen 64, 0379, Oslo, Norway

E-mail: [Marcin.Wojewodzic@kreftregisteret.no](mailto:Marcin.Wojewodzic@kreftregisteret.no)

^4)^ Institute of Marine Research, PB 6404, N-9294 Tromsø, Norway

E-mail: [thomas.bohn@hi.no](mailto:thomas.bohn@hi.no)

Tel. +47 97009916

*) The correspondence can be addressed to all authors

**Supplementary3 Information.**

**Supplementary3 Table 11.**

D. magna pre-miRNAs

>pre-miR-1_sc473_plus_1862-1922

CCGUGCUUCCUUACUUCCCAUAGUCAAAUGACAAACGAUAUGGAAUGUAAAGAAGUAUGGAG

>pre-miR-2a-1_sc24_minus_3279939-3280007

CUCGCAAAGUGGCUGUCAUGUGUCCCCCAAUAAUCAUGCGUUUCAUAUCACAGCCAGCUUUGAUGAGCG

>pre-miR-2a-2_sc24_minus_3279749-3279812

CUUGUCAGAGUGGUUGUGGUGUGUGUCUUUGUCGUACUCGUAUCACAGCCAGCUUUGAUGAGCG

>pre-miR-2b_sc24_minus_3280225-3280310

CGUCAAAGUCGACUGGGAAAUGCUGUAGAAAGAAUUCUCGCUAAUCCCGGAAUUUCGAAGACAUAUCACAGCCAGCUUUGACGAGC

>pre-miR-7_sc2190_plus_1222125-1222188

UGGAAGACUAGUGAUUUUGUUGUUCGGCAUAAAGACCGGUAACAAAAAAUCUCUAGUUCACCUA

>pre-mir-8_sc3025_minus_720090-720163

CAUCUUACCGGGCAGCAUUAGAUUGGCUUUGUAACAAGCACCAUGUGUUUCUAAUACUGUCAGGUAAAGAUGUC

>pre-miR-9a_sc3124_plus_175035-175095

UCUUUGGUUAUCUAGCUGUAUGACUGUUAGAAGACGUCAUAAAGCUAGGUUACCAAAGUUA

>pre-miR-9b_sc2190_minus_1745746-1745804

UCUUUGGUGGUCUAGCUGUAUGAGGUUUUUAAGUUCAUAAAGCUAGAUCAGCAAGGCAA

>pre-miR-10_co11822_plus_307-367

UACCCUGUAGAUCCGAAUUUGUAGUUGCAUCACAAGACAAAUUCGGUUCUAGAGAGGUUCG

>pre-miR-12_sc2703_minus_1623-1683

UGAGUAUUACAUCAGGUACUGGUUGUAUGUGCAUGGUACCAGUACCAUUGUAACACUCUCA

>pre-miR-13_sc24_minus_3280063-3280125

CGUCGGAAAGGCUGUGGUGUGUUGCUAUUACAUAAUUUUCAUAUCACAGCCAUUCUAGAUGCG

>pre-miR-31_sc1681_minus_3328-3389

AGGCAAGAUGUCGGCAUAGCUGACGUGUUAAUCCGAGUGUAGCUGUGUUACAUCGUGCCAAC

>pre-miR-33_sc248_minus_283491-283556

GUGCAUUGUAGUUGCAUUGCACGUUGUGAAUGAUUUGUUUCGAGCAAUGCUUCGGCAGUGCAACGC

>pre-miR-34_sc1361_plus_861121-861188

UGGCAGUGUGGUUAGCUGGUUGUGAAAUAUUUACUUCUGACUACACAGCCACUAUCCAAACUGUCCAC

>pre-miR-71_sc24_minus_3280380-3280445

UGAAAGACAUGGGUAGUGAGAUGUUGGCGUGGUAGUUCCACACCUCUCACUACUUUGUCGUUCACG

>pre-miR-87-1_sc1067_minus_2113-2176

AGCCUGAUUCUUGCUCGACCUAUGCUUCUAUCAAUGGGCGAGGUGAGCAAAGUUUCAGGUGCGU

>pre-miR-87-2_sc1067_minus_2294-2379

GGGCCUGAUCCUUUGCUCAACCUGUGAUCGUAAACCUAGUAUUACCCUCCUUGUGCGGGAGGAGGUGAGCAAAGUUUCAGGUGCGU

>pre-miR-92a_sc2957_plus_30615-30692

AGGCUUUGAUGGGUGUCAAUUUUUCGACUAAUUGCCAAAGAUGAAUGAGGCAGAAAUAUUGCACUCGUCCCGGCCUGU

>pre-miR-92b_sc2957_plus_30791-30850

AGGCUUAGGCAAGAGCAACUUCGUUCAUAUAUGCAUCGAAUUGCACUCGUCCCGGCCUGC

>pre-miR-100_sc243_plus_423404-423480

AACCCGUAGAUCCGAACUUGUGUGUGAAUUUGCAAAAAAAUCACAUCGUGCAUCACAAGUACGAUUCUAAGGGUCCA

>pre-miR-124_sc512_minus_3056358-3056420

CGUGUUCAUUGUUGUGCCUUUUGUAUUAUUGAACGGAAUCAUAAGGCACGCGGUGAAUGCCAA

>pre-miR-125_sc243_plus_424677-424739

UCCCUGAGACCCUAACUUGUGAGUGAUUGGAAUAAACUUAUCACAGGCUAGAUUCUCAGGUAU

>pre-miR-133_sc642_plus_1136823-1136887

AGCUGGCUGAAUCCGGGCCAAAUUGUUAUUCAUAGAGCAGCAUUUGGUCCCCUUCAACCAGCUGU

>pre-miR-137_sc2979_minus_99491-99552

GGGUAUUCUCGAGUGAUUAACACGUUAUGAAACCAGAGUUGUUAUUGCUUGAGAAUACACGU

>pre-miR-153_sc2486_minus_238053-238117

CUCAUUUUCGUGAUUUUUGCAAUUUGAAUAAACAAUUGGAAAUUGCAUAGUCACAAAAGUGAUGG

>pre-miR-184_sc3311_minus_6021-6118

CCUUAUCAUUCACCAGUCCGGUUAGAUUUUUUGAAAAAGUUCUCUUUCUUGGUCAUGCAAGAAUUACGAACUCUACUGGACGGAGAACUGAUAAGGGC

>pre-miR-190_sc24_minus_2298010-2298074

AGAUAUGUUUGAUAUUCUUGGUUGUUCCAUGUUCAGCCUACAACCAGAUGUCAGACAUAUUAUUA

>pre-miR-193_sc3334_minus_146109-146167

CGGGAUUUAGUGGAACAGUUGUUAUAUUAUGCAAAAAUACUGGCCUGCUAAGUCCCAAG

>pre-miR-210_sc1036_minus_60153-60228

AGCUGCUGAACACUGCCCAAGAUUGGCGUGAACAAUUCUAACGGAAGGCAACUCUUGUGCGUGUGACAGCGGCUAU

>pre-miR-219_sc560_plus_5726-5786

UGAUUGUCCAAACGCAAUUCUUGUGUAAAUACGACAACAAGAAAUGUGUGGGGACAUCGUU

>pre-miR-252a_sc2385_plus_1185121-1185186

UAAGUACUCGUGCCGCAGGAGAGAUGUAUUAAUCUGCAAGUUCCUCCUGCUGCUCAAGUUCUUAAC

>pre-miR-252b_sc512_plus_1309862-1309926

CUAAGUAGUAGUGCCGCAGGUAAUGGCUGUUAAAUAUCACGUUACCUGGACAAGGCUACUUAAUG

>pre-miR-263a_sc1361_minus_2445783-2446013

AAUGGCACUGGAAGAAUUCACGGGUUGCAUUUUUGUGUCAAUGAUUGGAAUUUGGUUUUGAUGGAGUCAAAAGAAGAGGCCGGAAGGAGUUUUUAUUUAUUUAUUUUUUAUUUUUUUUUUUUAAUUAUUAUUAUUACUUACUUCCAUUGAGGCCUCGCUUUAUUCUUUGUCUCUGAUCAAUUCUUUUCCAAUCUGAAAACAUUGAUACUCGUGGAUCUUCAGUGCUGUACC

>pre-miR-263b_sc1361_minus_2445501-2445563

CUUGGCACUGGAAGAAUUCACAGAGUGCAUUACGACAGGUCGUGGGUUCCCUGGUGCCAGAGA

>pre-miR-275_sc1361_minus_9029-9093

CGUGCUGCGACAGGUGCUUCCGGCUGUGUUAAUAUUUGCGAGUCAGGUACCUGAAGUAGCGCGCG

>pre-miR-276_sc389_plus_794239-794309

AGCGAGGUAUAGAGUUCCUACGUCUUGUCACUGAUUUCAAGUCGAUCGGUAGGAACUUCAUACCGUGCUCU

>pre-miR-277sc1361_plus_860062-860128

CGUACCAGCAGUGCGUUUGCCUUUAAAUAUUUGCCAAAGGGGGUAAAUGCAUUCUUCUGGUAUCGUC

>pre-miR-278_co12469_minus_467-620

CCGGACAAGAGUCUUUCACCGGCCGUGCCCAUUUUCGAACGAUUUUCCAGUUUCUCGGCGGCGGAGGAUCUUACUUCUUUUUUUUAAUUGAGAUUCCGUUUUCGAGAGACGGGAAUGUCUAAACAAAACAGGUCGGUGGGACUUUCGUCCGUGU

>pre-miR-279a_sc1005_plus_681513-681584

GAUGAUUGUGUGUCUGGUCCAUGUUAACUUUAUUUGGCAUUCGACAUUCAUGACUAGAUCCACACUCAUCCA

>pre-miR-279b_2190_minus_1744801-1744864

GGCGAGUUCGGUUUUAGUUAUAUUUUAUAUUCAGUCAGUAUGACUAGAACCCACACUCGUCCGG

>pre-miR-279c_sc2385_minus_1033185-1033249

GGUGGGUCUGUUUCUAGUGCAUGUGUUUUCUGCCUGUUUGUUCAUGACUAGAUCCAUACUCAUCU

>pre-miR-279d_sc2385_minus_1033657-1033733

GGUGGAUAUGGUUCUCGUGACAUGGCUUUUAUCGGCUUUGAUUUCCGAGUGCACAUGACUAGAUCCAUACUCACCAG

>pre-miR-279e_sc2190_minus_1745878-1745953

GAUGAGUGAGGAUCGUUAGUCGCGUUGAAAAAGUUCCUCAAGUUAAGAGUUCGUGACUAGAUCUGACACUCGUCCA

>pre-miR-281_sc1579_plus_315029-315096

AAGAGAGCUAUCCGUGAACAGUAUUGCUGUUUGUGCGAAAAUUCACUGUCAUGGAGCUGCUCUCUUUA

>pre-miR-282_sc1839_minus_8071-8157

UAGCCUCUCCUAGGCUUUGUCUUGUUGACGUUUGGGUUCUUGGCUUCGGCAAAAGAAAUUCUAGACAACGUCUUGGAGAGCGCUACU

>pre-miR-283_sc1551_minus_399-484

AAAUAUCAGCAGGUAAUUCUGGGAAAUAUUAUCCUUGAACUUGUUUCAAAUACAAGGAAGAACCCAGACUGCCGACUGGUAUCAGG

>pre-miR-285_sc2569_minus_184684-184751

ACUGAAUUCUUUUGAUGCCUAGAUGCAGUUUUUCUGACUAGAAUUCUAGCACCAUUGGAAUUCAGUUU

>pre-miR-305_sc1361_minus_8573-8660

AUUGUACUUCAUCAGGUGCUCUGGUCAAUCGUACGUAAACGUUUCCUUUGUGGAAAUCAUAUUCCCGGCACCUGCUGGAGUGCAAUUG

>pre-miR-307_sc3326_minus_606695-606785

CCUCGUUCAAUUUGGUUGUGGUGUAGUUUGUAAACAAAUCUGAUCAAAUCGAUCACUUUCUGUUUUCGUCACAACCUCCUUGAGUGAGUGA

>pre-miR-309_sc548_minus_1861-1920

AGGUAAACUUCGCCCAGAUUAGACUUAAUUAUUGCAGUCACUGGGUAAAGUUUGUCCGUU

>pre-miR-315_sc1265_plus_32184-32278

UUUUGAUUGUUGCUCAGAAAGCCGUGCCAUUUCGCUAUUGUUUGGCUGAACGCCGAAAACAUAUACAAACUGGCUUUCGAGAAAUAAUCAGAAUC

>pre-miR-317_sc1361_plus_859057-859121

GGGGAACCACCCUGCGUUCCCCUGUUACUUGAAUUAUCCAGGUGAACACAGCUGGUGGUAUCUCA

>pre-miR-375_sc2190_plus_706587-706827

ACUCGAGCUGAUCGUAUAAGCCUGGAGUUUUUUAUCGUGUUUUGUGACACGAGAAAAGAUUUCUCCAACUUCUCUGGCAGUCUUUUUCUUUUUUUUCUCUCUUUUUUCUUCUCGCUUUACUCCAAACUAUAAGAUGAUUGCCAUCUUCCAAUUGAGAGAAGGGUGAGAGGAAGAAAGGGAGGGACGUCAGCAGUUGUUGCGGUCACAUUUUCGAUUCGGUUUGUUCGUUCGGCUUGAGUUA

>pre-miR-745_sc1005_plus_846660-846724

CGGUCCUUCCCGGGGCACCUUGCCGUUAGGUAGUUGAGCAAGCGAGCUGCCCAGUGAAGGGCUUU

>pre-miR-750_sc243_minus_73854-74005

AGUUGGAAGUGGGGAUCUCGGCAUUUUUGUUCCUGGUUUCAAAAAAGGUGAGCCGGCGUUUCGGGAUUUGCUGUUGUAUUGAACGGCCAAGUUUCGAACGUCGAGCCAACCUUCAUAAUCGUUAUUUUGCCAGAUCUAUCUCUUCCAGCUCA

>pre-miR-965_sc2190_minus_1698674-1698737

AGGGAAAGGUUAUAGCUCUUAUGUGUCUUAUUUUGAUUACCAUAAGCGUAUGGCUUUUCCCCUG

>pre-miR-981_sc3124_minus_104984-105042

CGGGUUUCGCGACGCUCGAACGCCGUUUUACAGUCCGUUCGUUGUCGACGAAACCUGCA

>pre-miR-993_sc810_plus_3483-3547

CUACCCUGUAGCUCCGGGCUUUUGUUUCGUUUCUAGUAAUCAGAAGCUCGUUUCUACAGGUAUCU

>pre-miR-998_sc2190_minus_1745456-1745530

GCUGAGUCUUGUGGUUGAUGUGCUAGAUAUUUUAGCUUCAACUCUCGGUUGCAUAGCACCACGGGAUUCAGCCGC

>pre-miR-1175_sc243_minus_73661-73726

AAGUGGAGCAGUGGAUCUCCACUUUGAUACAUAAGAUAUAAGUGAGAUUCAACUACUCCAACUUCU

>pre-miR-2944_sc2190_minus_1745587-1745679

UAGGAACUACCGCUUGUGGUUGAUUGAAUUGUCGAUUUUUUAAAUUCUAAUCGAUUCAAUUGUCUUAUUUCUAUCACAGUCGUAGUUACUAGA

<pre-miR-3791_sc2190_minus_1744660-1744720

CGGUGAAUUCGACCAUGGUUUUGUUGAUUUAGAGAAACAUCACCGGGUAGAAUUCAUCCAG

>pre-miR-iab-4_sc7_minus_650154-650210

ACGUAUACUGAAUGUAUCCUGAGCUUCAUUCAUUCCGGUAUACCUUCAGUAUACGUA

>pre-miR-iab-8_sc7_plus_650153-650213

UUACGUAUACUGAAGGUAUACCGGAAUGAAUGAAGCUCAGGAUACAUUCAGUAUACGUCCA

>pre-bantam_sc2190_minus_20074-20136

CUAGUUUUCUCAGUGAUCUGCCAGAUAUUAUUUCAAUUUCUGAGAUCAUUGUGAAAGCUGAUU

>pre-let-7_sc243_plus_423613-23780

UGAGGUAGUAGGUUGUAUGGUUCGGUAUUACACAGCCGCGUUGCCGGAUAGACUCUAAAAAAGAUUGGACAAACCGGAUGGGUAAUUCCAUUUCGGUUCCGUUUCUUUCUUUUUGUUGAGAACAAAUGGCGCGCGCCACGGUGGAACUGUACAACUUGCUAACUUUCC

>pre-miR-nov-1_sc243_minus_73895-73959

GGUGAGCCGGCGUUUCGGGAUUUGCUGUUGUAUUGAACGGCCAAGUUUCGAACGUCGAGCCAACC

>pre-miR-nov-3_sc548_minus_2085-2145

UCUUGGUUGCUCGGUCUUUAGGAUUUUUAUCAUCAUUCCUAAAGCUCGGCUAGCAGGAUCC

>pre-miR-nov-4_sc548_minus_1700-1757

CCAGUUUAACAUAGCCCACAGAUUAUGUACAUUUCUCUGGGUUAUGAUUAAGACUGGG

>pre-miR-nov-10_sc1036_minus_421101-421163

UGAAGCAGAGGACUGCUUUGAAAAUGUCAUCUAUUAUCUUUGAGAGCAGUUCUCUGCUUCAUU

>pre-miR-nov-12_sc2190_minus_1745310-1745371

GGGGGGAACUUUACUCAGUUUGAUUCAUUUAAUUUGAAAGUCACUGGGUACGUUCGCCCUUG

>pre-miR-nov-2_sc548_minus_2233-(2295)

GACACUACAUGUAUCUAGUAUGACCAGUUAUACGGAGUCACUGGGUAUUCAUUGUAGGGUCGA

>pre-miR-nov-5_sc642_minus_169265-169330

GUGAGCUUGGAUUUAAAAGAAAUUCAUUGGGUUAUCCAUAAUAUUCUUUUUUUUCUCCACUCACAG

>pre-miR-nov-6_sc781_minus_364257-364319

AAUGUGAUAUUUUAUCCUUCUAAAAUUGCUAAUUAUUACUUUAGAAGGAUAAAAUAUCACAUU

>pre-miR-nov-7_sc1036_minus_1020892-(1020954)

ACUUCUGCUGGUUAGUGGUAACAUUGAUUACGUUUAAACGUUGCACUAACCGGUAGUGGGACG

>pre-miR-nov-8_sc1036_minus_1020730-(1020788)

ACCUUGAGCAGCCAAUUGCAUAGUAGCUUUAUUUCUUUGCACUGGCCUGCCCAGGGGCG

>pre-miR-nov-9_sc1036_minus_1020540-(1020602)

ACUUCGAAACGCCCAGUUGCUUUGUGGCAUCAAGACUUCAUUGCACUGUACGGUUCGAGGCGA

>pre-miR-nov-11_sc1036_minus_(420885)-420940

UUUAAAAUCUGACUGAGAGGUUUUUUAUAUCAGCCUCUCAGAUUGAUUUUAAAAAA

>pre-miR-nov-13_2703_minus_(2031)-2147

AAAUCUCAUACGGUAAUUUGGAAAAGAAUGGGGAAAUUUCUUGAUGGAAAUGGUUUUUCGCGCACCUGCGUGAUUUUCCAAAACCCAGUACCCGAAUUGCCGUCUGGGGUGGCCAGG

**Table 11**. The pre-miRNA sequences of the 66 conserved and 13 novel miRNA in *D. magna*.
